# Connecting the dots: Illusory pattern perception predicts belief in conspiracies and the supernatural

**DOI:** 10.1002/ejsp.2331

**Published:** 2017-09-25

**Authors:** Jan‐Willem van Prooijen, Karen M. Douglas, Clara De Inocencio

**Affiliations:** ^1^ Vrije Universiteit Amsterdam The Netherlands; ^2^ The NSCR The Netherlands; ^3^ The University of Kent United Kingdom

**Keywords:** Illusory pattern perception, conspiracy beliefs, supernatural beliefs, irrational beliefs

## Abstract

A common assumption is that belief in conspiracy theories and supernatural phenomena are grounded in illusory pattern perception. In the present research we systematically tested this assumption. Study 1 revealed that such irrational beliefs are related to perceiving patterns in randomly generated coin toss outcomes. In Study 2, pattern search instructions exerted an indirect effect on irrational beliefs through pattern perception. Study 3 revealed that perceiving patterns in chaotic but not in structured paintings predicted irrational beliefs. In Study 4, we found that agreement with texts supporting paranormal phenomena or conspiracy theories predicted pattern perception. In Study 5, we manipulated belief in a specific conspiracy theory. This manipulation influenced the extent to which people perceive patterns in world events, which in turn predicted unrelated irrational beliefs. We conclude that illusory pattern perception is a central cognitive mechanism accounting for conspiracy theories and supernatural beliefs.

People often hold irrational beliefs, which we broadly define here as unfounded, unscientific, and illogical assumptions about the world. Although many irrational beliefs exist, belief in conspiracy theories and belief in the supernatural are particularly prevalent among ordinary, nonpathological citizens, and are frequent topics of scientific research (Oliver & Wood, 2014; Sunstein & Vermeule, 2009; Swami et al., 2013; Wiseman & Watt, 2006). Conspiracy theories are commonly defined as the assumption that a group of people colludes together in secret to attain evil goals (e.g., Zonis & Joseph, 1994). While conspiracies can and do occur, and hence not all conspiracy theories are irrational (e.g., Watergate; The Iran‐Contra‐Affair), many conspiracy theories that citizens believe are unlikely in light of logic or scientific evidence, including theories that 9–11 was an inside job, that the pharmaceutical industry deliberately spreads diseases, or that climate change is a lie fabricated by scientists. Supernatural beliefs are defined as beliefs that violate scientifically founded principles of nature, including superstition, belief in the paranormal, horoscopes, and telepathy (Lindeman & Aarnio, 2007).

Such irrational beliefs are not necessarily harmless. Belief in conspiracy theories predicts maladaptive perceptions and behaviors such as withdrawal from politics, decreased civic virtue, hostility, and radicalization (Abalakina‐Paap, Stephan, Craig, & Gregory, 1999; Goertzel, 1994; Jolley & Douglas, 2014a, 2014b; Swami et al., 2011; Swami, Chamorro‐Premuzic, & Furnham, 2010; Van Prooijen, Krouwel, & Pollet, 2015). Supernatural beliefs may lead people to consult spiritual healers instead of qualified medical specialists to treat dangerous illnesses, or to base important life decisions (e.g., whether to buy a house, or get a divorce) on information derived from horoscopes or a random draw of tarot cards (Asser & Swan, 1998; Ernst, 2000; Mazur, 2008; Nahin, Barnes, Stussman, & Bloom, 2009; Shermer, 2011). These considerations suggest that it is important to investigate how such beliefs originate in the human mind. In the present research we investigate the overarching cognitive basis of the many types of irrational beliefs that people hold.

It has frequently been suggested that irrational beliefs are rooted in pattern perception, that is, the automatic tendency to make sense of the world by identifying meaningful relationships between stimuli (Zhao, Hahn, & Osherson, 2014). This is a functional process, as it enables people to recognize basic patterns that are real, and that are important to internalize (e.g., a red traffic light signals danger; drinking water quenches one's thirst; being unfriendly to a stranger may elicit an unfriendly response). Sometimes, however, there are distortions to this otherwise functional process as people may connect dots that are in fact unrelated, leading to illusory pattern perception—misperceiving meaningful patterns in what are in fact random stimuli.

Such illusory pattern perception emerges because people often have difficulty recognizing when stimuli do or do not occur through a random process. For instance, truly random sequences typically display less variation—and hence form more clusters—than people intuitively expect, creating the feeling of meaningful patterns that in fact occurred at random (Falk & Konold, 1997). Put differently, a random process often generates sequences that appear nonrandom to the human mind, and that may even contain occasional symmetries or esthetic regularities. As a result, it is difficult for people to appreciate the role of coincidence in generating these pattern‐like sequences (Williams & Griffiths, 2013). But whereas co‐occurring stimuli may be generated through either a nonrandom (i.e., actual patterns) or a random process, only nonrandomly generated stimuli can be considered meaningful as they have actual predictive value for what future stimuli the same process will generate. Illusory pattern perception occurs when people mistakenly perceive randomly generated stimuli as causally determined through a nonrandom process, and hence as diagnostic for what future stimuli to expect.

A common assumption, then, is that illusory pattern perception is at the core of many of the irrational beliefs that people hold (e.g., Shermer, 2011; Wiseman & Watt, 2006; Whitson & Galinsky, 2008). Given how fundamental and widely accepted this assumption is, however, it is surprising how little direct empirical evidence there is available to support the role of illusory pattern perception in irrational beliefs in general, and particularly in the domain of conspiracy theories. The current program of research is designed to fill this void.

## Illusory Pattern Perception and Irrational Beliefs

In numerous life domains, people make predictions of future outcomes by searching for patterns in random stimuli. For instance, illusory pattern perception shapes people's expectations during sports matches. In a study of the “hot hand in basketball”, both spectators and players believed that the chance of players hitting a shot was related to the success of their previous shot. In reality, the relationship between hits and misses of succeeding shots by the same player did not statistically deviate from chance (Gilovich, Vallone, & Tversky, 1985). Related to this, habitual gamblers are more likely than non‐habitual gamblers to detect patterns in random stimuli (Wilke, Scheibehenne, Gaissmaier, McCanney, & Barrett, 2014). Even pigeons seem subject to illusory pattern perception. In a classic study by Skinner (1948), hungry pigeons received food at regular time intervals, and as a result, the pigeons increasingly started doing whatever they were doing the last time that they received food. As noted by Skinner, “The experiment might be said to demonstrate a sort of superstition. The bird behaves as if there were a causal relation between its behavior and the presentation of food, although such a relation is lacking” (p. 171).

Pattern perception thus enables people to predict an uncertain future by ‘connecting the dots’ and establishing meaningful relations between stimuli. Such predictions of future outcomes are also inherent in irrational beliefs, which are frequently construed as ways to cope with uncertain and complex life situations. One common proposition is that belief in conspiracy theories often constitutes attempts to understand distressing events that are difficult to understand otherwise (Hofstadter, 1966; see also Bale, 2007). Consistently, impactful and threatening societal events increase people's sense‐making motivation—as reflected in feelings of worry and a desire to find out what happened—which subsequently increases belief in conspiracy theories (Van Prooijen & Van Dijk, 2014). In a similar vein, supernatural beliefs help people to make sense of their life and to predict the future. Supernatural beliefs have been argued to imbue the world with meaning and purpose, and therefore help people cope with the basic uncertainties that are inherent to life (e.g., Mazur, 2008; Shermer, 2011). These arguments suggest that irrational beliefs help people make sense of their world by increasing a subjective sense of predictability, and pattern perception is a key element of this process.

The desire to make sense of the world is of particular importance to people when they lack control (Park, 2010) or when they are uncertain (Van den Bos, 2009). Consistently, empirical findings reveal that people are particularly likely to believe conspiracy theories when they lack control or are uncertain (Newheiser, Farias, & Tausch, 2011; Marchlewska, Cichocka, & Kossowska, in press; Sullivan, Landau, & Rothschild, 2010; Van Prooijen, 2016; Van Prooijen & Acker, 2015; Van Prooijen & Jostmann, 2013; for a review, see Kossowska & Bukowski, 2015). Likewise, lacking control or experiencing feelings of uncertainty have been found to increase supernatural beliefs, in the form of superstition (Whitson & Galinsky, 2008), belief in horoscopes (Wang, Whitson, & Menon, 2012), and increased religiosity (Hogg, Adelman, & Blagg, 2010; Kay, Gaucher, McGregor, & Nash, 2010). These findings are consistent with the idea that irrational beliefs are rooted in pattern perception, as establishing relevant patterns makes an unpredictable, uncertain, and potentially threatening environment more predictable. Indeed, control threats have been found to increase the extent to which people misperceive patterns in randomness, and these findings closely mirrored the effects of control threats on irrational beliefs in consecutive experiments (Van Harreveld, Rutjens, Schneider, Nohlen, & Keskinis, 2014; Whitson & Galinsky, 2008). These studies render further plausibility to the idea that pattern perception and irrational beliefs are grounded in a similar psychological function, namely, to imbue the world with order.

Various complementary lines of research are consistent with the general idea that irrational beliefs are related to a tendency to misperceive patterns in randomness. For instance, conspiracy beliefs are correlated with constructs such as paranormal beliefs (Barron, Morgan, Towell, Altemeyer, & Swami, 2014; Darwin, Neave, & Holmes, 2011; Lobato, Mendoza, Sims, & Chin, 2014; Newheiser et al., 2011; Swami et al., 2011), the tendency to attribute agency and intentionality where it does not exist (Douglas, Sutton, Callan, Dawtry, & Harvey, 2016; Imhoff & Bruder, 2014), and the conjunction fallacy (Brotherton & French, 2014; for illustrations of the relationship of the conjunction fallacy with paranormal belief, see Rogers, Davis, & Fisk, 2009; Rogers, Fisk, & Wiltshire, 2011). Although these studies indicate links between conspiracy theories and a variety of heuristics and cognitive biases, and are therefore suggestive of a link between pattern perception and conspiracy belief, direct evidence is currently lacking. Indeed, one recent study tested the relationship of conspiracy beliefs with people's subjective perception of randomness in binary strings that varied in their actual level of complexity. This study found no relationship between conspiracy beliefs and subjective randomness (Dieguez, Wagner‐Egger, & Gauvrit, 2015).

The relationship between illusory pattern perception and supernatural beliefs has been tested more extensively, and results are suggestive for such a relationship. For instance, Valdesolo and Graham (2014) found that supernatural beliefs were associated with agency detection, that is, the belief that events are caused intentionally by purposeful agents. Furthermore, supernatural beliefs correlate with a tendency to misperceive patterns in randomness (e.g., Blackmore & Trościanko, 1985; Bressan, 2002; Brugger, Landis, & Regard, 1990; Dagnall, Parker, & Munley, 2007; Musch & Ehrenberg, 2002; for a review, see Wiseman & Watt, 2006). Also for supernatural beliefs, however, this association does not emerge in all studies (Roberts & Seager, 1999) and under all circumstances (Blagrove, French, & Jones, 2006; Bressan, 2002). In the present research, therefore, our aim was to expand on these insights through a program of research designed to test the assertion that irrational beliefs—both in conspiracies and the supernatural—are empirically connected with a tendency to perceive patterns in randomly generated stimuli.

## Research Overview

In the present research, we conducted five studies in which we focused on the relationship between irrational beliefs and illusory pattern perception. Consistent with previous approaches, in Study 1 we first tested if conspiracy beliefs and supernatural beliefs are correlated with a tendency to perceive patterns in randomly generated coin toss outcomes (cf. Dagnall et al., 2007). In Study 2, we manipulated whether participants searched for patterns in these coin toss outcomes, and tested whether this predicts irrational beliefs through an increase in pattern perception. In Study 3, we focused on pattern perception in visual stimuli (see also Blackmore & Moore, 1994), and examined whether pattern perception necessarily needs to be *illusory* to predict irrational beliefs. In Study 4, we manipulated whether participants read a text supporting either paranormal beliefs, conspiracy theories, or skepticism, and we tested whether agreement with these texts predicted pattern perception. Finally, in Study 5, we manipulated people's belief in a specific conspiracy theory (cf. Jolley & Douglas, 2014a, 2014b), and tested if this predicts an increased tendency to perceive patterns in the world, belief in unrelated conspiracy theories, and supernatural beliefs.

## Study 1

As a first test we developed a measure of pattern perception that was based on a randomly generated string of coin toss outcomes. Specifically, we assessed whether or not participants detected patterns in random coin toss outcomes, and tested the correlations of such pattern perception with irrational beliefs.

### Method

#### Participants and design

This study had a cross‐sectional design, and was run online through the Crowdflower forum—a website for crowdsourcing that closely resembles Amazon's Mechanical Turk. The studies reported in this article were programmed in Qualtrics such that each IP‐address could participate only once. A total of 264 US participants completed the study (89 men, 175 women; *M*_*age*_ = 36.61 years, *SD* = 12.07). The study took about 15 to 20 minutes to complete, and participants were awarded a small payment (0.75 USD).

#### Measures

We assessed belief in conspiracy theories with two complementary measures. First, we asked for participants' agreement with a series of well‐known conspiracy theories that are frequent topics of discussion on conspiracy websites (e.g., Douglas & Sutton, 2011; Van Prooijen et al., 2015). We specifically asked participants to indicate the degree to which they believed nine statements are true (1 = *definitely not true*, 5 = *definitely true*), for instance “The US government deliberately conceals a lot of information from the public”, and “The US government had advance knowledge of the 9/11 attacks”. These nine items were averaged into a reliable belief in existing conspiracy theories scale (*α* = .87) (full materials, including all scale items, are in the Supporting Materials).

Second, we measured belief in a fictitious (i.e., experimenter‐designed) conspiracy theory. This measure complements the existing conspiracy belief measure by soliciting responses that have not been influenced by peers, the Internet, or social media. We assessed an abbreviated (i.e., 9‐item) and adapted version of Swami et al.’s (2011) Red Bull conspiracy theory measure. Example items are “Red Bull contains illegal substances that raise the desire for the product”, and “The official inventor of Red Bull pays 10 million Euro each year to keep food controllers quiet” (1 = *definitely not true*, 5 = *definitely true*). These items were averaged into a reliable measure of belief in fictitious conspiracy theories (*α* = .92)

To measure supernatural beliefs, participants responded to the validated, 30‐item magical ideation scale (Eckblad & Chapman, 1983), which we adapted by asking participants to respond to the items on a scale ranging from 1 (*strongly disagree*) to 5 (*strongly agree*). Example items are “I think I could learn to read other people's minds if I wanted to”, “Horoscopes are right too often for it to be a coincidence”, and “Numbers like 13 and 7 have no special powers” (reverse‐coded). Participants' responses to these items were averaged into a reliable supernatural belief scale (*α* = .93)

To develop a measure of pattern perception, we used the website https://www.random.org to create a random coin toss 100 times, with the only predetermined restriction being 50 Heads and 50 Tails (i.e., the expected distribution in a random process). Then, we subdivided the full sequence into 10 separate sets of 10 consecutive coin tosses. For each set, participants were asked to rate the extent to which they believed the sequence was fully random, or fully determined. Responses were on a scale ranging from 1 (*completely random*) to 7 (*completely determined*). Examples of coin sequences were “HTHHTTTTHH” and “HHHTTTTTHH”. After the 10th sequence, participants were asked to answer the following question on the same rating scale: “Now, imagine that the above items represent 100 consecutive throws with the same coin. Please again rate how random or determined the outcomes are”. Together, the ratings of the 10 sets and the rating of the full sequence formed a reliable 11‐item pattern perception scale (*α* = .95). 1Consistent with the notion that it is often hard to distinguish random sequences from sequences that truly emerged through a nonrandom process (Williams & Griffiths, 2013), our first string presented to participants emerged by chance as symmetrical (HTHHHHHHTH). We therefore also analyzed the correlations of our final item—asking whether the full string of 100 coin tosses was random—with irrational beliefs. Results further supported the hypothesis that our three measures of irrational beliefs are correlated with a tendency to perceive patterns in random coin toss outcomes (.23 < *r*s < .33, *p*s < .001). At the end of the study, participants were thanked and debriefed.

### Results and Discussion

The results are displayed in Table 1. Perceiving patterns in randomly generated coin toss outcomes was significantly correlated with both measures of conspiracy beliefs, and with supernatural beliefs (*r*s > .36, *p*s < .001). These findings are the first to directly suggest a relationship between belief in conspiracy theories and pattern perception, and conceptually replicate this relationship for supernatural beliefs. Furthermore, these findings suggest that participants indeed displayed substantial variation in the extent to which they detect patterns in the random coin toss outcomes, and hence, we use these coin tosses as the basis for our experimental manipulation in Study 2.

**Table 1:** Means, Standard Deviations, and Intercorrelations of the Measured Variables —Study 1

	*M*	*SD*	1	2	3	4
1. Belief in existing conspiracy theories	2.88	0.84	‐			
2. Belief in fictitious conspiracy theories	2.69	0.84	.70***	‐		
3. Supernatural beliefs	2.46	0.69	.47***	.54***	‐	
4. Illusory pattern perception	2.82	1.51	.37***	.44***	.38***	‐

*** *p <* .001.

## Study 2

In Study 2, we utilized the random coin outcome sequences that we tested in Study 1 to manipulate pattern search. In order to guess the next coin outcome following each sequence, participants either were, or were not, instructed to look for patterns in the coin tosses. As the cognitive process of pattern perception tends to occur particularly when people predict uncertain outcomes (Gilovich et al., 1985; Wilke et al., 2014), we reasoned that intuitively searching for patterns in random sequences as a means to guess the next coin toss would increase the likelihood of people perceiving patterns in the coin toss sequences. We therefore predicted that searching for patterns in random sequences would increase irrational beliefs through an increase in pattern perception.

### Method

#### Participants and design

The study had a design with two conditions (intuitive pattern search: high vs. low), and was run online through the Crowdflower forum on a US sample. A total of 223 people participated (72 men, 103 women, 48 not indicated; *M*_age_ = 36.01 years, *SD* = 11.88). The study took about 15 to 20 minutes, and participants were rewarded with a small payment (0.75 USD).

#### Procedure

The study was presented as consisting of two parts. In the first part, participants were asked to play a “coin tossing game”. They saw the outcomes of 10 sequences of 10 coin tosses—these sequences were identical to those used in the pattern perception measure of Study 1. The first of these sequences was presented as an example; the “real” coin tossing game consisted of the remaining nine sequences.

For each sequence, participants' task was to guess what the next coin outcome would be (Heads or Tails). Within this context, we manipulated intuitive pattern search. In the high pattern search condition, participants received the following instruction before starting the game: “Try to see if you can find a pattern in each sequence. Do NOT try to calculate this—use your intuition. Ask yourself: ‘Do I see a pattern here—and based on that, what next coin outcome would make most sense?’” In the low pattern search condition, participants received the following instruction: “These are random sequences, generated by the website http://random.org. In a particular sequence there may be more Heads or Tails; this is to be expected when a sequence is random. Each coin toss is independent and has an exact probability of 50% of being a Head or a Tail.” After completing the game, we assessed participants' pattern perception with the following item: “To what extent were the coin flip sequences random, or showed a pattern?” (1 = *they were totally random*, 7 = *they totally showed a pattern*). Furthermore, we assessed participants' current mood on a slider ranging from 1 (*very negative*) to 100 (*very positive*) as a filler task and also as a means to test whether the effects of the intuitive pattern search manipulation are attributable to mood effects.

Participants then started the second part of the study, in which they responded to a series of statements. Here, we measured belief in existing conspiracy theories (*α* = .84), belief in fictitious conspiracy theories (*α* = .86), and supernatural beliefs (*α* = .94) with the same scales as in Study 1. Upon completion of the questionnaire, participants were thanked and debriefed.

### Results and Discussion

Means, standard deviations, and intercorrelations of the study variables are reported in Table 2.

**Table 2:** Means, Standard Deviations, and Intercorrelations of the Measured Variables —Study 2

	*M*	*SD*	1	2	3	4	5
1. Belief in existing conspiracy theories	2.84	0.77	‐				
2. Belief in fictitious conspiracy theories	2.70	0.74	.60***	‐			
3. Supernatural beliefs	2.24	0.70	.49***	.52***	‐		
4. Illusory pattern perception	3.67	1.71	.23**	.29***	.32***	‐	
5. Mood	68.83	18.59	.07	.14	.00	.20**	‐

** *p* < .01;

*** *p <* .001.

#### Pattern perception

An ANOVA on the pattern perception measure revealed a significant effect of the intuitive pattern search manipulation, *F*(1, 179) = 8.26, *p =* .005; η^2^ = .04. Participants in the high pattern search condition detected clearer patterns in the random sequences (*M* = 4.03, *SD* = 1.64) than participants in the low pattern search condition (*M* = 3.32, *SD* = 1.71). These findings indicate that the manipulation successfully influenced the extent to which participants perceived patterns in the coin toss sequences.

#### Irrational beliefs

A MANOVA on the three dependent variables yielded no significant multivariate or univariate effects, all *F*s < 1. Contrary to predictions, the intuitive pattern search manipulation did not exert a direct effect on the dependent variables.

As noted above, however, the manipulation did influence the extent to which participants perceived patterns in the coin toss outcomes. Furthermore, consistent with Study 1, we found that the pattern perception measure was significantly correlated with belief in existing conspiracy theories (*r* = .23, *p* = .002), belief in fictitious conspiracy theories (*r* = .29, *p* < .001), and magical ideation (*r* = .32, *p* < .001). Given that we predicted the manipulation to influence irrational beliefs because of its effects on people's tendency to see patterns in the sequences, we tested the indirect effect of the intuitive pattern search manipulation (effect‐coded: 1 high pattern search, −1 low pattern search) on irrational beliefs through pattern perception. As indicated by the fact that 0 was not in the 95% confidence interval, bootstrapping analyses (5000 samples) utilizing the “MEDIATE” macro by Hayes and Preacher (2014) revealed a significant indirect effect on all three dependent variables: for belief in existing conspiracy theories, (*B* = 0.04, *SE* = 0.02) CI_95%_[0.01; 0.09], for belief in fictitious conspiracy theories (*B* = 0.05, *SE* = 0.02) CI_95%_[0.02; 0.11], and for supernatural beliefs (*B* = 0.05, *SE* = 0.02) CI_95%_[0.01; 0.10]. These findings suggest that whereas the intuitive pattern search manipulation did not exert a direct effect on irrational beliefs, it did exert an indirect effect on all three dependent variables through pattern perception.

Although the lack of a direct effect precludes conclusions about causality, the findings of the present study suggest that intuitively searching for patterns in the coin toss sequences increases pattern perception, which in turn predicts irrational beliefs. As such, the indirect effect that we observed in Study 2 further supports a role for pattern perception in belief in conspiracy theories and supernatural beliefs.

#### Mood

The intuitive pattern search manipulation did not influence participants' mood, *F* < 1. Hence, the results relating to irrational beliefs are not attributable to variations in participants' mood.

## Study 3

Studies 1 and 2 are consistent with the theoretical notion that pattern perception is a central aspect of irrational beliefs. These findings are restricted, however, by the fact that all coin toss sequences were random, and there was no base‐rate comparison with nonrandom sequences. Moreover, it has been noted that randomly generated binary sequences are often difficult to distinguish from sequences that were truly determined (Williams & Griffiths, 2013). As such, there are two interpretations possible for these findings. The first interpretation is that pattern perception in general predicts irrational beliefs. Specifically, irrational beliefs may be associated with a generalized tendency to detect patterns, rendering strong believers more likely to perceive both illusory *and* real patterns. The implication of this is that clearer perception of patterns predicts irrational beliefs regardless of whether these patterns are real or illusory. A second interpretation, however, is that pattern perception necessarily needs to be illusory to predict irrational beliefs. According to this interpretation, it is specifically perception of patterns in random or chaotic stimuli that predict irrational beliefs—not perception of real patterns.

In Study 3 we examined these competing interpretations by focusing on visual stimuli, that is, modern art paintings. In particular, some of our participants evaluated modern art paintings that arguably contain patterns by displaying a meaningfully ordered structure. Other participants evaluated paintings that were highly chaotic and arguably do not contain patterns, as the paint strokes on canvas appear largely random. These latter paintings allow for illusory pattern perception, as some people may start perceiving nonrandom figures in the paint strokes. Assuming that participants see patterns more clearly in the structured than in the chaotic paintings, the two competing interpretations render the following possibilities. If general pattern perception (i.e., regardless of whether they are real or illusory patterns) predicts irrational beliefs, then such beliefs should increase to the extent that people perceive patterns more clearly. Alternatively, if illusory pattern perception predicts irrational beliefs, then only perceiving patterns in chaotic paintings should be associated with such beliefs, not detecting the existing patterns in the structured paintings.

### Method

#### Participants and Design

We tested our line of reasoning in a design with two conditions (Modern art paintings: structured paintings vs. unstructured paintings). The study was run online through Crowdflower, on a US sample. A total of 214 participants completed the study (87 men, 118 women, 9 not indicated; *M*_age_ = 35.00 years, *SD* = 11.43). Again, the study lasted 15 to 20 minutes, and participants received a small payment (0.75 USD).

#### Procedure

The study was introduced as consisting of two parts. The first part was about “evaluating modern art paintings”. Participants were informed that they would evaluate a total of nine modern art paintings, all by the same artist. We then manipulated whether participants saw structured or chaotic modern art paintings. In the structured paintings condition, participants were informed that they would see paintings by an artist that is “well‐known for his regular design and alignment of figures”, and subsequently evaluated nine paintings by the Hungarian Artist Victor Vasarely (the name of the painter was not disclosed to participants in either condition). We anticipated that most participants would see clear patterns in these relatively structured paintings. In the unstructured paintings condition, participants were informed that they would see paintings by an artist that is “well known for his random brush strokes and irregular figures”, and subsequently evaluated nine paintings by the US artist Jackson Pollock. We anticipated that most participants would not see clear patterns in these relatively chaotic paintings (see the Supporting Materials for the Vasarely and Pollock paintings that were presented to participants). 2Art specialists may differ in opinion on the question if, and to what extent, Pollock's paintings actually are “random” or “chaotic”. More relevant for the present research, however, is how participants subjectively perceived these paintings. As reported in the Results section, the Means on the pattern perception measure reveal that participants on average saw little structure in these paintings, as intended.


In both conditions, we asked three questions after each painting. The first two questions were designed to disguise the true purpose of the research from participants, and as possible control variables in the analyses: “How ugly or beautiful do you find this painting?” (1 = *very ugly*, 7 = *very beautiful*), and “How familiar are you with this painting?” (1 = *never seen before*, 7 = *very familiar*). The third question was “To what extent do you see a pattern in this painting? (If you only see random strokes of paint, answer “1”; if you clearly see a pattern, answer “7″)” (1 = *not at all*, 7 = *very much*). These ratings were averaged into reliable 9‐item scales of beauty (*α* = .92), familiarity (*α* = .97), and pattern perception (*α* = .97). After evaluating all the paintings, we asked participants to indicate their current mood on a slider ranging from 1 (*very negative*) to 100 (*very positive*).

Then, participants started the second part of the study, which was about “personal beliefs”. In this part of the study, we again measured belief in existing conspiracy theories (*α* = .84), belief in fictitious conspiracy theories (*α* = .90), and supernatural beliefs (*α* = .93) with the same scales as in the previous studies. At the end of the study, participants were thanked and debriefed.

### Results and Discussion

The Means, standard deviations, and intercorrelations of the study variables are displayed in Table 3.

**Table 3:** Means, Standard Deviations, and Intercorrelations of the Measured Variables —Study 3

	*M*	*SD*	1	2	3	4	5	6	7
1. Belief in existing conspiracy theories	2.81	0.75	‐						
2. Belief in fictitious conspiracy theories	2.66	0.75	.55***	‐					
3. Supernatural beliefs	2.12	0.68	.50***	.49***	‐				
4. Pattern perception	4.28	1.99	−.01	.07	−.08	‐			
5. Perceived beauty	3.84	1.30	.11	.16*	.19**	.49***	‐		
6. Perceived familiarity	1.64	1.21	.38***	.32***	.56^***^	.11	.33	‐	
7. Mood	72.93	17.91	.03	.11	.00	.15*	.32***	.02	‐

* *p* < .05;

** *p* < .01;

*** *p <* .001.

#### Pattern perception

An ANOVA on the pattern perception measure revealed a significant effect of the modern art paintings manipulation, *F*(1, 204) = 311.23, *p* < .001; η^2^ = .60. Participants clearly saw patterns in the structured Vasarely paintings (*M* = 5.82, *SD* = 1.08), but they did not clearly see patterns in the unstructured Pollock paintings (*M* = 2.74, *SD* = 1.40), as intended with this manipulation.

#### General pattern perception as predictor of irrational beliefs

The first possible explanation for the previous findings is that perception of patterns in general—regardless if they are real or illusory—predicts stronger irrational beliefs. If this were true, one would expect that evaluating the structured Vasarely paintings, in which most people detect patterns, would increase these beliefs as compared to evaluating the unstructured Pollock paintings, which most people consider to be relatively chaotic and devoid of patterns.

A MANOVA on the dependent variables revealed a significant multivariate effect of the manipulation, *F*(3, 201) = 2.64, *p* = .050; η^2^ = .04. The univariate effect was significant for belief in existing conspiracy theories, *F*(1, 203) = 6.84, *p* = .010, η^2^ = .03, and for supernatural beliefs, *F*(1, 203) = 3.89, *p* = .050; η^2^ = .02, but not for belief in fictitious conspiracy theories, *F*(1, 203) = 1.15, *p* = .284. Contrary to the idea that general pattern perception increases these beliefs, however, participants believed existing conspiracy theories more strongly (*M* = 2.95, *SD* = 0.79), and reported stronger supernatural beliefs (*M* = 2.22, *SD* = 0.67) after evaluating the unstructured Pollock paintings than after evaluating the structured Vasarely paintings (for belief in existing conspiracy theories, *M* = 2.67, *SD* = 0.70; for supernatural beliefs, *M* = 2.03, *SD* = 0.68). Thus, the manipulation exerted an effect that was *opposite* to the idea that irrational beliefs are grounded in general pattern perception (i.e., including perception of truly existing patterns). Instead, these findings are consistent with previous findings that ambiguous stimuli increase conspiracy beliefs (Van Harreveld et al., 2014).

Furthermore, in the overall sample the pattern perception measure was uncorrelated with belief in existing conspiracy theories (*r* = −.01, *p* = .92), with belief in fictitious conspiracy theories (*r* = .07, *p* = .33), and with magical ideation (*r* = −.08, *p* = .28), revealing no evidence for a mediational role of pattern perception in general. Taken together, these findings do not support the idea that pattern perception predicts irrational beliefs regardless of whether the patterns are illusory, or widely detected by others.

#### Illusory pattern perception as predictor of irrational beliefs

We then tested the second possible explanation for the previous findings, which is that only *illusory* pattern perception predicts irrational beliefs. If this were true, then one would expect that only perceiving patterns in the unstructured, chaotic Pollock paintings predicts irrational beliefs—and not recognizing the widely detected patterns in the structured Vasarely paintings. To test this line of reasoning, we computed the correlations of pattern perception with both measures of conspiracy beliefs and supernatural beliefs within each modern art painting condition separately. 3We decided not to base our conclusions on the interaction term between the modern art painting manipulation and the continuous pattern perception measure, given the strong direct effect of the manipulation on pattern perception. Nevertheless, it is noteworthy that this interaction term was highly significant for belief in existing conspiracy theories (*β* = −.17, *p* = .017) and magical ideation (*β* = −.32, *p* < .001), and marginally significant for belief in fictitious conspiracy theories (*β* = −.14, *p* = .057).


Consistent with the idea that irrational beliefs are driven by illusory pattern perception only, perceiving patterns in the unstructured Pollock paintings significantly predicted belief in existing conspiracy theories (*r* = .36, *p* < .001), belief in fictitious conspiracy theories (*r* = .31, *p* = .002), and supernatural beliefs (*r* = .32, *p* = .001). Recognizing patterns in the highly structured Vasarely paintings, however, was unrelated to belief in existing conspiracy theories (*r* = .00, *p* = .98) and belief in fictitious conspiracy theories (*r* = .03, *p* = .77), and *negatively* predicted supernatural beliefs (*r* = −.31, *p* = .002). These correlations differed significantly between conditions, as indicated by Fisher r‐to‐z tests (for belief in existing conspiracy theories, *z* = 2.64, *p* = .008; for belief in fictitious conspiracy theories, *z* = 2.03, *p* = .042; for supernatural beliefs, *z* = 4.59, *p* < .001).

These findings suggest that only perceiving patterns in random or chaotic stimuli (i.e., illusory pattern perception) predicts irrational beliefs, and not recognizing patterns in structured stimuli. Belief in conspiracy theories was unrelated to perception of these existing patterns, and supernatural beliefs even predicted a decreased capacity to recognize existing patterns.

#### Perceived beauty

An ANOVA on perceived beauty revealed a significant effect of the modern art painting manipulation, *F*(1, 204) = 10.83, *p* = .001; η^2^ = .05. Participants found the structured paintings by Vasarely more beautiful (*M* = 4.13, *SD* = 1.16) than the unstructured paintings by Pollock (*M* = 3.55, *SD* = 1.38). Controlling for perceived beauty in the main analyses of the dependent variables did not change any of the reported effects, however.

#### Perceived familiarity

An ANOVA on perceived familiarity did not reveal a significant effect of the manipulation, *F* < 1. Average ratings on familiarity were very low on the scale (*M* = 1.64, *SD* = 1.21), suggesting that participants were not familiar with the paintings. Intriguingly, ratings of familiarity were strongly correlated with irrational beliefs (see Table 3), suggesting that such beliefs may be grounded in a general tendency to overestimate one's knowledge—a possibility that future research may explore further.

#### Mood

An ANOVA revealed no significant effect of the modern art painting manipulation on participants' mood, *F* < 1 (*M* = 72.93, *SD* = 17.91). Effects of the manipulation can thus not be attributed to participants' mood.

## Study 4

Studies 2 and 3 manipulated pattern perception and measured irrational beliefs as dependent measures. The results of these two studies were consistent with the correlational findings from Study 1. In Study 4, we aimed to find further support for this relationship by treating pattern perception as the dependent measure. Specifically, we manipulated whether participants read a short text written by a paranormal believer, a conspiracy theorist, or a skeptic, with various indicators of pattern perception as dependent measures. We specifically tested whether participants' agreement with the paranormal and conspiracy texts, but not their agreement with the skeptic text, predicted their tendency to perceive patterns. The study measured three indicators of pattern perception, including the coin toss measure (Study 1) and the extent to which participants detected patterns in Jackson Pollock's paintings (Study 3). As a third indicator we also assessed the extent to which participants perceive patterns in world events, that is, participants' belief that many events in the world co‐occur not through coincidence but through a nonrandom process.

### Method

#### Participants and design

The study had three conditions (paranormal belief; conspiracy belief; skeptic). We recruited a total of 455 US participants online at the Crowdflower forum, of whom 401 participants completed the study (171 men, 228 women, 2 not indicated; *M*_age_ = 35.93, *SD* = 12.28). The study lasted about 15 minutes, and participants received a small payment (0.75 USD).

#### Procedure

Participants first read a short excerpt that had ostensibly been taken from someone's Internet blog. We manipulated whether the writer of the blog believed paranormal phenomena, conspiracy theories, or whether the writer was skeptical about these issues. For instance, in the paranormal condition, the blog started: “I believe that there are hidden forces of nature that people do not understand yet, and that determine many important events in life. . .”. In the conspiracy condition, the blog started: “I believe that there are hidden organizations that influence citizens' lives in ways that people do not understand, and that explain many events that occur in society. . .”. In the skeptic condition, the blog started: “I do not believe in mysterious natural forces or secret organizations. Although we may not always know everything, by and large people have a good sense of how the world works. . .” (full texts in the Supporting Materials). After the blog, we asked whether participants agreed with the writer (1 = *Not at all*, 7 = *Very much*), after which participants were asked to describe a situation where hidden forces of nature seemed at work (supernatural condition), a conspiracy seemed at work (conspiracy condition), or events emerged coincidentally (skeptic condition) to reinforce the manipulation. We also assessed participants' mood on a slider (1 = *Very negative*, 100 = *Very positive*).

As dependent measures we assessed three indicators of pattern perception. First, we assessed the extent to which participants perceive patterns in world events (1 = *Strongly disagree*, 7 = *Strongly agree*): “Societal events that seem unrelated frequently are in fact related”, “Many things that happen in the world are no coincidence”, and “There is a grain of truth in the saying that the wings of a butterfly can cause a hurricane elsewhere”. We averaged these items into an indicator of pattern perception for world events (*α* = .68). Furthermore, we assessed the extent to which participants saw patterns in the Jackson Pollock paintings used in Study 3, and averaged participants' responses into a reliable scale (patterns in paintings; *α* = .92). Finally, we assessed the same coin toss measure as in Study 1 (patterns in coin tosses; *α* = .90). After this, participants were thanked and debriefed.

### Results and Discussion

The means, standard deviations, and intercorrelations of the measured variables are displayed in Table 4. We analyzed the results with hierarchical regression analyses. First, we coded the conditions as two orthogonal contrasts designed to test our line of reasoning (Paranormal vs. Conspiracy vs. Skeptic, Contrast 1: −1 ‐1 2; Contrast 2: 1–1 0). Furthermore, we mean‐centered the measure of participants' agreement with the writer. We entered the two contrasts and participants' agreement with the writer in Step 1. We then added the interactions of the two contrasts with agreement in Step 2. Degrees of freedom deviated from the total sample due to attrition during the study.

**Table 4:** Means, Standard Deviations, and Intercorrelations of the Measured Variables – Study 4

	*M*	*SD*	1	2	3	4	5
1. Patterns in life	4.32	1.22	‐				
2. Patterns in paintings	2.93	1.42	.16**	‐			
3. Patterns in coin tosses	2.61	1.24	.15**	.33***	‐		
4. Agreement	3.39	1.18	.33***	.18***	.15**	‐	
5. Mood	65.58	21.43	.19***	.02	−.07	.11*	‐

* *p* < .05;

** *p* < .01;

*** *p* < .001.

#### Pattern perception

The regression results are displayed in Table 5. Step 1 was significant for all three indicators of pattern perception: patterns in life, (*R* ^*2*^ = .12) *F*(3, 406) = 18.60, *p* < .001; patterns in paintings, (*R* ^*2*^ = .03) *F*(3, 402) = 4.64, *p* = .003; patterns in coin tosses, (*R* ^*2*^ = .04) *F*(3, 398) = 5.22, *p* = .002. The main effect of agreement was significant for all three pattern perception indicators. More importantly, we also found a significant main effect of the first contrast—comparing the paranormal and conspiracy conditions vs. the skeptic condition—on two out of three pattern perception indicators (Patterns in life and patterns in coin tosses; see Table 5). Participants perceived more patterns in life in the paranormal (*M* = 4.38, *SD* = 1.22) and conspiracy (*M* = 4.31, *SD* = 1.30) conditions than in the skeptic condition (*M* = 4.24, *SD* = 1.13). Likewise, participants perceived more patterns in coin tosses in the paranormal (*M* = 2.62, *SD* = 1.24) and conspiracy (*M* = 2.74, *SD* = 1.20) conditions than in the skeptic condition (*M* = 2.48, *SD* = 1.29). The second contrast—comparing the paranormal vs. conspiracy conditions—was nonsignificant for all pattern perception measures. These findings suggest that only reading about paranormal or conspiracy beliefs is sufficient to cause a slight increase in pattern perception.

**Table 5:** Results of Hierarchical Regression Analyses – Study 4

	Patterns in life		Patterns in paintings		Patterns in coin tosses	
*Step 1*	*B(SE)*	*t*(406)	*B(SE)*	*t*(402)	*B(SE)*	*t*(398)
Contrast 1	−0.08(0.04)	−2.06*	0.02(0.05)	0.41	−0.10(0.04)	−2.17*
Contrast 2	−0.02(0.07)	−0.25	−0.04(0.09)	−0.51	−0.09(0.08)	−1.13
Agreement	0.36(0.05)	7.41***	0.21(0.06)	3.57***	0.19(0.05)	3.57***
*Step 2*	*B(SE)*	*t*(404)	*B(SE)*	*t*(400)	*B(SE)*	*t*(396)
Contrast 1 x Agreement	−0.25(0.03)	−7.32***	−0.08(0.04)	−1.70^†^	−0.20(0.04)	−5.41***
Contrast 2 x Agreement	0.04(0.05)	0.76	0.11(0.07)	1.59	−0.10(0.06)	−1.69†

† *p* < .10;

* *p* < .05;

*** *p* < .001.

More importantly, Step 2 was significant for patterns in life (Δ*R* ^*2*^ = .10) *F*(2, 404) = 26.87, *p* < .001, and for patterns in coin tosses (Δ*R* ^*2*^ = .07) *F*(2, 396) = 16.45, *p* < .001, and it was marginal for patterns in paintings (Δ*R* ^*2*^ = .01) *F*(2, 400) = 2.59, *p* = .076. Likewise, the crucial contrast 1 x agreement interaction was highly significant for patterns in life and patterns in coin tosses, and marginal for patterns in paintings (see Table 5). The contrast 2 x agreement interaction was nonsignificant for patterns in life and patterns in paintings, and marginal for patterns in coin tosses.

**Figure 1:**
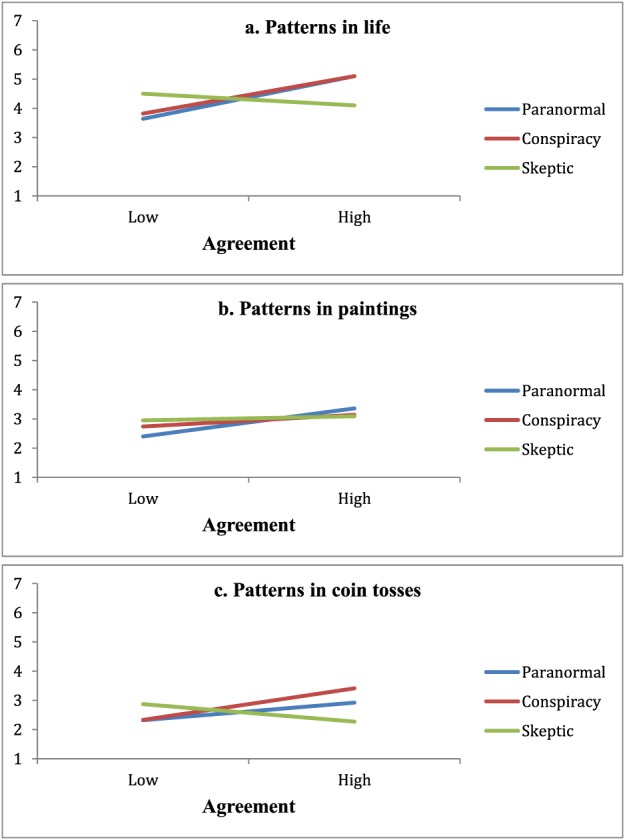
**(A–C)** The Relationships of Agreement with Pattern Perception—Study 4 [Colour figure can be viewed at http://wileyonlinelibrary.com]

We then examined the contrast 1 x agreement interaction by calculating the correlations of agreement with the pattern perception measures within each condition. Agreement with the paranormal blog correlated positively with pattern perception (patterns in life, *r* = .59, *p* < .001; patterns in paintings, *r* = .31, *p* < .001; patterns in coin tosses, *r* = .24, *p* = .006), as did agreement with the conspiracy theory blog (patterns in life, *r* = .52, *p* < .001; patterns in paintings, *r* = .16, *p* = .070; patterns in coin tosses, *r* = .48, *p* < .001). Agreement with the skeptic blog, however, correlated negatively with patterns in life (*r* = −.16, *p* = .065) and patterns in coin tosses, (*r* = −.21, *p* = .014) and did not correlate with patterns in paintings (*r* = .05, *p* = .60). The relationships of agreement with the pattern perception measures in the three conditions are displayed graphically in Figures 1a–1c. These findings indicate that agreeing with paranormal beliefs and conspiracy theories, but not agreeing with a skeptic, predicts pattern perception.

#### Mood

In our analysis of the mood measure, Step 1 was significant, (*R* ^*2*^ = .02) *F*(3, 404) = 3.05, *p* = .028. The effect of Contrast 2 was significant (*B* = 2.68, *SE* = 1.29, *p* = .039) and the effect of agreement was marginal (*B* = 1.76, *SE* = 0.91, *p* = .053). Step 2 was nonsignificant, (Δ*R* ^*2*^ = .01) *F*(2, 402) = 1.87, *p* = .16. The crucial contrast 1 x agreement interaction can hence not be attributed to participants' mood.

## Study 5

Studies 1 to 4 all support the idea that irrational beliefs are related with illusory pattern perception. These findings emerged when manipulating pattern search, with irrational belief as dependent measure (Studies 2 and 3) as well as when manipulating whether participants read about irrational beliefs, with pattern perception as dependent measure (Study 4). In Study 5, we assessed the theoretical and practical implications of the relationship between irrational beliefs and pattern perception. Specifically, we examined whether pattern perception constitutes an explanation for the frequently observed relationships between conceptually unrelated irrational beliefs.

A common research finding is that belief in one conspiracy theory predicts belief in other, unrelated conspiracy theories. This finding is usually interpreted as evidence that acceptance of one conspiracy theory reinforces a more general belief system assuming that the world is being governed by conspiracies (i.e., a “monological belief system”, or a “conspiratorial mindset”; see Goertzel, 1994; Douglas & Sutton, 2011; Lewandowsky, Oberauer, & Gignac, 2013; Swami et al., 2010, 2011, 2013; Van Prooijen et al., 2015; Wood, Douglas, & Sutton, 2012; but see Sutton & Douglas, 2014). What we find problematic about this interpretation, however, is that acceptance of a conspiracy theory is also a strong predictor of non‐conspiratorial forms of belief. A robust finding in irrational belief research is that conspiracy beliefs and supernatural beliefs are strongly intercorrelated (Barron et al., 2014; Darwin et al., 2011; Lobato et al., 2014; Newheiser et al., 2011; Swami et al., 2011), a finding that we replicated in the current research (see Tables 1, 2, 3).

Previous research offers various explanations for the link between conspiracy theories and supernatural beliefs, such as anti‐conformist tendencies and an inclination to reject conventional explanations or authority opinions (e.g., Swami et al., 2011). We propose an additional explanation for this relationship: A conspiracy theory often describes specific meaningful relationships between world events that perceivers assume to have taken place (e.g., the conspiracy theory that democratic bankers caused the financial crisis to get Obama elected; or, the conspiratorial inferences drawn from the observation that Donald Rumsfeld happened to be in the opposite side of the Pentagon when the plane hit the building on 9/11/2001). Put differently, acceptance of a conspiracy theory implies an increase in the extent to which people perceive patterns in world events, as reflected in the belief that instead of being a coincidence, many events that happen in the world are somehow causally related. This perception of patterns in world events is associated with other, unrelated irrational beliefs. Consistently, it has been suggested previously that supernatural beliefs are rooted in a failure to appreciate that events often co‐occur by coincidence (Blackmore & Trościanko, 1985).

To test this line of reasoning, in Study 5 we manipulated belief in a conspiracy theory through a validated procedure (cf. Jolley & Douglas, 2014a, 2014b). While in Study 4 participants read a rather broad text about a person believing conspiracy theories, in Study 5 participants read an Internet excerpt either underscoring or undermining the validity of a specific conspiracy theory by presenting focused arguments. We predicted that this conspiracy theory manipulation would increase the extent to which people generally believe events in the world to be somehow related (i.e., pattern perception of world events), which in turn predicts conceptually unrelated conspiracy beliefs and supernatural beliefs.

### Method

#### Participants and design

We ran the study online via the Crowdflower website on a US sample. The design had two conditions, one in which participants read an article supporting conspiracy theories (pro‐conspiracy condition) and one in which participants read an article not supporting conspiracy theories (anti‐conspiracy condition). The study lasted about 15 minutes. There were 228 participants (95 men, 119 women, 14 missing; *M*_age_ = 35.91 years, *SD* = 11.80) who received a small payment for participation (0.75 USD).

#### Procedure

Participants read a fragment from a purported Internet article in which we manipulated information about conspiracy theories. We adapted the manipulation from Jolley and Douglas (2014a) but tailored it towards a US context. In the pro‐conspiracy condition, the article described that there are many good reasons to be suspicious of governmental operations. Subsequently, the article specifically highlighted the NSA surveillance program, emphasizing how the NSA keeps track of communication between US citizens on a widespread scale; and, the 9/11 attacks, emphasizing that there are many inconsistencies in the official accounts. In the anti‐conspiracy condition, the article described that there are very few reasons to be suspicious of governmental operations. Then, the article emphasized how the NSA surveillance program essentially is a computer algorithm to detect suspicious activity, designed to ensure citizens' safety. Also, the article described that as to the 9/11 attacks, there is no evidence to support other accounts than the official one (the exact wording can be found in the Supporting Materials).

To check the manipulation, we asked the following questions (1 = *not at all*, 7 = *very much*): “Is there reason for concern about the NSA security programs?”, “Is there reason to think that the NSA listens to private phone conversations that are unrelated to terrorist plots?”, “Is there reason to think that the NSA reads the content of e‐mail and Internet chat messages that are unrelated to terrorist plots?”, and “Is there reason to be suspicious about other governmental operations besides the NSA's surveillance programs?”. These items were averaged into a reliable manipulation check scale (*α* = .91). In addition, we again measured participants' mood on a slider (1 = *very negative*, 100 = *very positive*).

We then assessed the extent to which people perceive patterns in world events with the same measure as in Study 4 (*α* = .68). After this, we measured belief in existing conspiracy theories unrelated to those varied in the experimental manipulation. Given the reference to the US government in the manipulation, we selected only the five items from the belief in existing conspiracy theories scale assessed in the previous studies that made no explicit reference to the US government (i.e., the items about Ebola, global warming, oil companies, the moon landing, and HIV/AIDS; *α* = .81). 4If we analyzed the full 9‐item scale including the items referring to the US government, results were similar. Specifically, the effect of the conspiracy manipulation was significant, *F*(1, 214) = 8.01, *p* = .005; η^2^ = .04, as was the indirect effect through pattern perception for world events (*B* = 0.05, *SE* = .03) CI_95%_[.01; 0.11]. Furthermore, we again assessed belief in fictitious conspiracy theories (*α* = .90) and supernatural beliefs (*α* = .94) utilizing the same scales as in the previous studies. At the end of the study, participants were thanked and debriefed.

### Results and Discussion

The means, standard deviations, and intercorrelations of the measured variables are displayed in Table 6.

**Table 6:** Means, Standard Deviations, and Intercorrelations of the Measured Variables—Study 5

	*M*	*SD*	1	2	3	4	5	6
1. Belief in existing conspiracy theories	2.51	0.89	‐					
2. Belief in fictitious conspiracy theories	2.72	0.78	.61***	‐				
3. Supernatural beliefs	2.29	0.72	.59***	.55***	‐			
4. Manipulation check	4.38	1.70	.18**	.16*	.07	‐		
5. Pattern perception for world events	4.55	1.18	.36***	.43***	.23**	.38***	‐	
6. Mood	67.11	19.77	.11	.09	.11	.06	.13	‐

* *p* < .05;

** *p* < .01;

*** *p <* .001.

#### Manipulation check

An ANOVA revealed stronger belief in a NSA conspiracy theory in the pro‐conspiracy condition (*M* = 5.35, *SD* = 1.27) than in the anti‐conspiracy condition (*M* = 3.34, *SD* = 1.48), *F*(1, 214) = 115.15, *p* < .001; η^2^ = .35. These results indicate that the manipulation worked as intended.

#### Pattern perception for world events

On the scale measuring the extent to which participants saw patterns in world events, the conspiracy manipulation exerted a significant effect albeit with a small effect size, *F*(1, 213) = 5.35, *p* = .022; η^2^ = .03. Participants perceived more patterns in world events in the pro‐conspiracy condition (*M* = 4.73, *SD* = 1.10) than in the anti‐conspiracy condition (*M* = 4.36, *SD* = 1.25). These results support the idea that conspiracy theorizing increases the perception of patterns in world events.

#### Irrational beliefs

We then analyzed the three dependent variables with a MANOVA. The multivariate effect was marginal, *F*(3, 211) = 2.24, *p* = .085; η^2^ = .03, and the univariate effect was only significant for belief in existing conspiracy theories, *F*(1, 213) = 5.72, *p* = .018; η^2^ = .03 (pro‐conspiracy condition *M* = 2.64, *SD* = 0.89; anti‐conspiracy condition *M* = 2.36, *SD* = 0.86), and not for belief in a fictitious conspiracy theory (*F* < 1) or supernatural beliefs, *F*(1, 213) = 1.05, *p* = .31; η^2^ = .005.

Further testing revealed, however, that the three dependent variables all were significantly correlated with the measure of pattern perception in world events (for belief in existing conspiracy theories, *r* = .36, *p* < .001; for belief in a fictitious conspiracy theory, *r* = .43, *p* < .001; and for supernatural beliefs, *r* = .23, *p* < .001). Consistent with our line of reasoning, we therefore proceeded to test the indirect effect of the conspiracy manipulation on our dependent variables through pattern perception in world events. Bootstrapping analyses (5000 samples) utilizing the MEDIATE macro (Hayes & Preacher, 2014) revealed a significant indirect effect for all three dependent variables: for belief in existing conspiracy theories (*B* = 0.05, *SE* = 0.02) CI_95%_[0.01; 0.10]; for belief in a fictitious conspiracy theory (*B* = 0.05, *SE* = 0.02) CI_95%_[0.01; 0.11]; and for supernatural beliefs (*B* = 0.03, *SE* = 0.01) CI_95%_[0.004; 0.06]. These findings reveal that being exposed to a conspiracy theory increased the extent to which people perceive patterns in world events, which in turn predicts a range of unrelated irrational beliefs. This finding supports our line of reasoning.

#### Mood

There was no effect of the conspiracy manipulation on participants' mood, *F*(1, 216) = 1.45, *p* = .23; η^2^ = .01. The effects of the manipulation on world pattern perception and the dependent variables are thus not attributable to participants' mood.

## General Discussion

Although people hold many different conspiracy and supernatural beliefs, psychological theories have assumed such beliefs to be rooted in largely similar underlying cognitive processes, specifically illusory pattern perception—that is, a tendency to perceive meaningful patterns in stimuli that were actually generated through a random process (Shermer, 2011; Whitson & Galinsky, 2008; Wiseman & Watt, 2006). Given how fundamental this assumption is within this research domain, it is surprising to find how little direct evidence exists for this assertion, particularly in the context of conspiracy theories. In the present study, our aim was to offer firmer empirical grounds for the role of pattern perception in irrational beliefs. Study 1 revealed significant correlations between conspiracy beliefs, supernatural beliefs, and a tendency to perceive patterns in randomness. In Study 2 we manipulated participants' intuitive pattern search, and results revealed an indirect effect such that intuitive pattern search predicted pattern perception, which in turn predicted irrational belief. Study 3 focused on visual stimuli. We found that only seeing patterns in chaotic stimuli predicted irrational beliefs, and not detecting patterns in structured stimuli. In Study 4, we manipulated whether participants read either a paranormal, conspiracist, or skeptic blog, and results revealed that only agreement with the paranormal and conspiracist blogs positively predicted pattern perception. Finally, in Study 5 we tested how pattern perception connects conceptually unrelated beliefs. Following a manipulation of belief in one conspiracy theory, people saw events in the world as more strongly causally connected, which in turn predicted unrelated irrational beliefs. Taken together, these findings support the assumption that illusory pattern perception is a basic cognitive aspect of the conspiracy and supernatural beliefs under investigation here.

One might note that randomly generated stimuli sometimes produce sequences that appear to contain actual patterns. Indeed, some of our coin toss sequences contain relatively long chunks of the same outcome (Heads or Tails), making it difficult for people to discriminate between real and illusory patterns (Williams & Griffiths, 2013). We propose, however, that this is precisely one reason why people hold irrational beliefs. People often fail to appreciate how likely it is that a random process generates stimuli that appear nonrandom (Falk & Konold, 1997). As a consequence, people tend to underestimate the likelihood that the patterns they perceive occurred through a random process. In a similar vein, people often encounter co‐occurring events in their daily life that appear nonrandom or purposeful, but that in fact were entirely coincidental (e.g., thinking of an old friend who then suddenly calls). The difficulty of distinguishing between sequences that were generated through a random vs. nonrandom process mirrors the difficulty of distinguishing between events that did vs. did not co‐occur through coincidence.

At first blush, the present findings seem inconsistent with a study that did not find significant correlations between pattern perception in binary outcomes and conspiracy beliefs (Dieguez et al., 2015). Closer inspection suggests two possible ways in which these diverging findings may be integrated by future research. As a first observation, the binary outcomes presented to participants in the study by Dieguez and colleagues (strings of 12) were not produced by a random generator, but instead were chosen to vary in their level of complexity. Indeed, one of their research goals was to assess how well subjective randomness correlates with actual randomness, and correspondingly, some of their strings were highly unlikely to occur through a random process (e.g., a string with 12 times the same outcome). We speculate here that people who are good at detecting randomness are also likely to be good at detecting nonrandomness. Hence, their measure of subjective randomness combined misperception of patterns in random sequences with correct detection of patterns in nonrandom sequences, within the same score. It is conceivable that the inclusion of nonrandom sequences suppressed correlations with conspiracy beliefs.

As a second observation, at least in two of the studies by Dieguez et al. (2015), participants were undergraduate students (in their third study, education level of participants was not reported). Previous research found that probability biases and paranormal beliefs are correlated in general population samples but not in university samples (Blagrove et al., 2006; Bressan, 2002), suggesting that the relationship between illusory pattern perception and irrational beliefs does not emerge in the highly educated strata. This is consistent with the observation that people with high education levels, or with strong analytic thinking skills, are less susceptible to irrational beliefs than people with low education levels or weak analytic thinking skills (Aarnio & Lindeman, 2005; Douglas et al., 2016; Gervais & Norenzayan, 2012; Musch & Ehrenberg, 2002; Swami, Voracek, Stieger, Tran, & Furnham, 2014; Van Prooijen, 2017). Clearly, more research is needed to fully establish the conditions under which pattern perception does, and does not, predict irrational beliefs. Our research suggests, however, that it would be premature to dismiss illusory pattern perception as a cognitive mechanism underlying irrational beliefs on the basis of only one set of studies that did not find evidence for this mechanism.

### Limitations and Future Research

In Study 2, and for some of the dependent variables in Study 5, we only found an indirect effect, and not a direct effect of the manipulation on irrational beliefs. We suspect that the relatively large base‐rate variance in these variables precludes a direct influence of relatively subtle manipulations in between‐subjects designs. Specifically, from the outset people differ substantially in how strongly they endorse conspiracy theories and supernatural beliefs, with some people being convinced skeptics and others being highly susceptible to such beliefs. People are also likely therefore to vary significantly in their sensitivity to subtle experimental manipulations. Although the lack of a direct effect precludes strong conclusions about cause and effect, one should bear in mind that the main purpose of this research was to illuminate the extent to which illusory pattern perception is part of the underlying cognitive processes that support irrational beliefs. As the results reveal, illusory pattern perception was quite susceptible to the experimental manipulations, which subsequently accounted for people's irrational beliefs. These findings were highly robust across studies, and were observed on two different measures of conspiracy beliefs (i.e., real and fictitious conspiracy theories), and on an extensive and validated measure of supernatural belief (Eckblad & Chapman, 1983).

In the present contribution we predominantly focus on the cognitive similarities between conspiracy beliefs and supernatural beliefs. Illusory pattern perception is one of the processes that binds these types of irrational beliefs, and is likely part of the reason why these beliefs are strongly correlated (Barron et al., 2014; Darwin et al., 2011; Lobato et al., 2014; Newheiser et al., 2011; Swami et al., 2011). Study 5 empirically examined whether the relationship between unrelated irrational beliefs is attributable to the assumption that seemingly unrelated events in the world are causally connected (i.e., perceiving patterns in world events). One might argue that our measure of perceiving patterns in world events conceptually overlaps with the irrational beliefs under investigation here, as both conspiracy theories and supernatural beliefs inherently assume causal connections between world events. Note, however, that in Study 4 our measure of patterns in world events correlated significantly with other indicators of pattern perception, suggesting good construct validity (see Table 4). Furthermore, in Study 5 our measure of patterns in world events correlated moderately but not strongly with irrational beliefs (see Table 6; .22 < *r*s < .44), suggesting related but distinct constructs. Nevertheless, we regard the assumption that pattern perception mediates empirical relationships between conceptually unrelated irrational beliefs as preliminary, and more research is needed to further examine this issue.

Besides similarities, we should recognize that there are also qualitative differences between conspiracy beliefs and supernatural beliefs. Unlike supernatural beliefs, conspiracy beliefs typically have a clear intergroup dimension given that by definition a hostile outgroup (i.e., the conspiracy) is considered to be deceptive and threatening to one's ingroup (e.g., fellow citizens; Crocker, Luhtanen, Broadnax, & Blaine, 1999; Kramer & Schaffer, 2014; Van Prooijen & Van Lange, 2014). Moreover, the margin of error differs for both types of irrational beliefs. Whereas most supernatural beliefs are impossible given the laws of physics as we currently understand them, conspiracy theories often can at least theoretically be true—and sometimes conspiracies do occur (e.g., Watergate; the Tuskegee syphilis experiment; the Milli Vanilli entertainment fraud), which may reinforce other, less realistic conspiracy theories. Future research focusing on irrational beliefs may clarify not only their similarities, but also their differences in terms of underlying psychological processes.

### Concluding Remarks

It has frequently been noted that both conspiracy and supernatural beliefs are widespread among the population of normal, mentally sane adults (Lindeman & Aarnio, 2007; Oliver & Wood, 2014; Sunstein & Vermeule, 2009; Wiseman & Watt, 2006). Why are these irrational beliefs so widespread? In the present research, we addressed this question by focusing on the cognitive processes underlying irrational beliefs. The answer that emerges from our data is that irrational beliefs are associated with a distortion of an otherwise normal and functional cognitive process, namely, pattern perception. People need to detect existing patterns in order to function well in their physical and social environment; however, this process also leads them to sometimes detect patterns in chaotic or randomly generated stimuli. Whereas the role of illusory pattern perception has frequently been suggested as a core process underlying irrational beliefs, the actual evidence for this assertion hitherto was unsatisfactory. The present findings offer empirical evidence for the role of illusory pattern perception in irrational beliefs. We conclude that illusory pattern perception is a central cognitive ingredient of beliefs in conspiracy theories and supernatural phenomena.

## Supporting information

Supporting information
