# Supplementary material for: Connecting the dots: Illusory pattern perception predicts belief in conspiracies and the supernatural
Source: Eur J Soc Psychol. 2017 Sep 25;48(3):320–35. doi: 10.1002/ejsp.2331 (PMC5900972; doi:10.1002/ejsp.2331)
Supplement: Supplementary file 1 — Supporting information [file EJSP-48-320-s001.docx]

Online Supplementary Materials

For:

**Connecting the Dots:**

**Illusory Pattern Perception Predicts Belief in Conspiracies and the Supernatural**

Jan-Willem van Prooijen^1,2^, Karen Douglas^3^, & Clara De Inocencio^1^

^1^ VU Amsterdam

^2^ The NSCR

^3^ The University of Kent

**Study 1**

***Existing conspiracy belief scale*:**

There is often debate about whether or not the public is told the whole truth about various important issues. These questions are designed to assess your beliefs about some of these subjects. Please indicate the degree to which you believe each statement is likely to be true.

(1 = *definitely not true*, 5 = *definitely true*)

- The US government deliberately conceals a lot of information from the public

- Ebola is a man-made virus

- The US government had advance knowledge of the 9/11 attacks

- The US government covered up crucial information in the aftermath of J. F. Kennedy’s assassination

- The science behind global warming has been invented or distorted out of self-interest

- Various wars in the Middle East were launched by oil companies

- The moon landing was a hoax

- The HIV/aids virus has been genetically engineered to wipe out certain sectors of the population

- Evidence of unidentified flying objects and extraterrestrial visitors is being suppressed by the government

***Fictitious conspiracy belief scale (adapted from Swami et al., 2011)*:**

There is a debate about whether or not the public is told the whole truth about the healthiness of certain energy drinks. These questions are designed to assess your beliefs about Red Bull in particular. Please indicate the degree to which you believe each statement is likely to be true.

(1 = *definitely not true*, 5 = *definitely true*)

| - Red Bull contains illegal substances that raise the desire for the product |
| --- |
| - The official inventor of Red Bull pays 10 million Euros each year to keep food controllers quiet |
| - If a can of Red Bull is heated up to 104ºF, it releases health-threatening substances |
| - Subliminal messages in Red Bull television commercials make consumers believe that Red Bull improves one's health |
| - The slogan "Red Bull gives you wings" is used because in animal experiments, rats grew rudiment wings |
| - Regular consumption of Red Bull raises dopamine levels, which causes damage in the long term |
| - In the beginning, Red Bull was illegal for minors in America, which raises questions as to its subsequent legalization |
| - Commercials in sports give the impression that Red Bull is healthy |
| - The extract "testiculus taurus" found in Red Bull has unknown side effects |

***Supernatural beliefs (adapted from Eckblad & Chapman, 1983):***

Please, indicate the degree to which you agree with the following statements.

(1 = *strongly disagree*, 5 = *strongly agree*)

| - Some people can make me aware of them just by thinking about me |
| --- |
| - I have had the momentary feeling that I might not be human |
| - I have sometimes been fearful of stepping on sidewalk cracks |
| - I think I could learn to read other's minds if I wanted to |
| - Horoscopes are right too often for it to be a coincidence |
| - Things sometimes seem to be in different places when I get home, even though no one has been there |
| - Numbers like 13 and 7 have no special powers (R) |
| - I have occasionally had the silly feeling that a TV or radio broadcaster knew I was listening to them |
| - I have worried that people on other planets may be influencing what happens on earth |
| - The government refuses to tell us the truth about flying saucers |
| - I have felt that there were messages for me in the way things were arranged, like in a store window |
| - I have never doubted that my dreams are the products of my own mind (R) |
| - Good luck charms don't work (R) |
| - I have noticed sounds on my records that are not there at other times |
| - The hand motions that strangers make seem to influence me at times |
| - I almost never dream about things before they happen (R) |
| - I have had the momentary feeling that someone's place has been taken by a look­alike |
| - It is not possible to harm others merely by thinking bad thoughts about them (R) |
| - I have sometimes sensed an evil presence around me, although I could not see it |
| - I sometimes have a feeling of gaining or losing energy when certain people look at me or touch me |
| - I have sometimes had the passing thought that strangers are in love with me |
| - I have never had the feeling that certain thoughts of mine really belonged to someone else (R) |
| - When introduced to strangers, I rarely wonder whether I have known them before (R) |
| - If reincarnation were true, it would explain some unusual experiences I have had |
| - People often behave so strangely that one wonders if they are part of an experiment |
| - At times I perform certain little rituals to ward off negative influences |
| - I have felt that I might cause something to happen just by thinking too much about it |
|  |
| - I have wondered whether the spirits of the dead can influence the living |
| - At times I have felt that a professor's lecture was meant especially for me |
| - I have sometimes felt that strangers were reading my mind |

***Illusory pattern perception:***

**How random are these coin flips?**

Below you each time see the results of the same coin being flipped 10 times ("H" means Heads, and "T" means Tails). Please rate the extent to which you see a pattern. If you believe the coin flip results are completely random, answer "1". If you believe the coin flip results are completely determined (for instance because of a biased coin, or if the results were rigged), please answer "7".

(1 = *completely random*, 7 = *completely determined*)

| - HTHHHHHHTH |
| --- |
| - HTHHTTTTHH |
| - HHHTTTTTHH |
| - HTHHHTHTHH |
| - HTTHHTTTTT |
| - HTTHTHHHTT |
| - THHTTTHHTH |
| - HTHHHTHTHT |
| - TTHTTHTHHT |
| - TTHTTTTHTT |
| - Now, imagine that the above items represent 100 consecutive throws with the same coin. Please again rate how random or determined the outcomes are. |

**Study 2**

**Intuitive pattern search manipulation**

***High intuitive pattern search condition:***

**Coin tossing game**

Welcome to the first part of this study. In this part, you will play a coin tossing game. Each time, you will see the results of a coin being tossed for 10 consecutive times ("Heads" or "Tails"). Your task will be to guess the next coin toss outcome for each sequence. 

IMPORTANT: Try to see if you can find a pattern in each sequence. Do NOT try to calculate this--use your intuition. In each sequence, ask yourself: "Do I see a pattern here--and based on that, what next coin outcome would make most sense?"

At the end, we will ask you to estimate how many of your predictions you think were correct. At the end of the study, we will let you know how well you did.

On the next screen, we will give an example.

--*NEW SCREEN--*

Here is an example of a coin toss sequence ("H" means Heads, "T" means Tails). Your task is to guess the next coin outcome.

Again: Try to see if you can find a pattern in each sequence. Do NOT try to calculate this--use your intuition. Ask yourself: "Do I see a pattern here--and based on that, what next coin outcome would make most sense?"

H T H H H H H H T H

Based on the pattern that you see, what coin outcome would you expect next?

*Heads / Tails*

--*NEW SCREEN--*

On the next screen, you will start for real with the coin tossing game. There will be a total of 9 sequences.

--*NEW SCREEN—*

**Sequence 1**

Remember: Do NOT try to calculate the pattern--use your intuition. Ask yourself: "Do I see a pattern here--and based on that, what next coin outcome would make most sense?"

H T H H T T T T H H

Based on the pattern that you see, what coin outcome would you expect next?

*Heads / Tails*

--*NEW SCREEN--*

*The procedure was then repeated 8 times with the following coin sequences:*

| - HHHTTTTTHH |
| --- |
| - HTHHHTHTHH |
| - HTTHHTTTTT |
| - HTTHTHHHTT |
| - THHTTTHHTH |
| - HTHHHTHTHT |
| - TTHTTHTHHT |
| - TTHTTTTHTT |

***Low intuitive pattern search condition:***

**Coin tossing game**

Welcome to the first part of this study. In this part, you will play a coin tossing game. Each time, you will see the results of a coin being tossed for 10 consecutive times ("Heads" or "Tails"). Your task will be to guess the next coin toss outcome for each sequence. 

IMPORTANT: These are random sequences, generated by the website random.org. In a particular sequence there may be more Heads or Tails; this is to be expected when a sequence is random. Each coin toss is independent and has an exact probability of 50% of being a Head or a Tail.

At the end, we will ask you to estimate how many of your guesses you think were correct. At the end of the study, we will let you know how well you guessed.

On the next screen, we will give an example.

--*NEW SCREEN--*

Here is an example of a coin toss sequence ("H" means Heads, "T" means Tails). Your task is to guess the next coin outcome.

Again: Keep in mind, this is a random sequence, and regardless of the previous coin tosses, the next toss will have a 50% chance of being Heads or Tails.

H T H H H H H H T H

Now make a guess: Which coin outcome will be next?

*Heads / Tails*

*--NEW SCREEN—*

On the next screen, you will start for real with the coin tossing game. There will be a total of 9 sequences.

*--NEW SCREEN—*

**Sequence 1**

Remember: This is a random sequence, and regardless of the previous coin tosses, the next toss will have a 50% chance of being Heads or Tails.

H T H H T T T T H H

Now make a guess: Which coin outcome will be next?

*Heads / Tails*

--*NEW SCREEN--*

*The procedure was then repeated 8 times with the following coin sequences:*

| - HHHTTTTTHH |
| --- |
| - HTHHHTHTHH |
| - HTTHHTTTTT |
| - HTTHTHHHTT |
| - THHTTTHHTH |
| - HTHHHTHTHT |
| - TTHTTHTHHT |
| - TTHTTTTHTT |

***Measures:***

You will hear how many of your answers were correct at the very end of the Study. Before proceeding, please answer some questions about the coin toss game.

- To what extent were the coin flip sequences random, or showed a pattern? (1 = *they were totally random*, 7 = *they totally showed a pattern*)

Now, please indicate your **current mood** on this slider.

How would you describe your mood at this moment? (ranging from 0 = very negative, to 100 = very positive)

***Introduction second part of the study:***

In this second part of the study, you will be asked to read a number of statements. Some statements pertain to societal or political events, others pertain to certain beliefs you may or may not have in life. Note that there are no right or wrong answers; we just want to find out how you think and feel about the statements asked.

In sum, your task will be to rate the extent to which you believe in, or agree with several statements.

Click >> to start

*Measures of existing conspiracy beliefs, fictitious conspiracy beliefs, and supernatural beliefs were the same as in Study 1.*

**Study 3**

This study consists of two parts.

In this first part, your task will be to evaluate a series of modern art paintings, and to answer some questions about each painting. In the second part, you will be asked your personal beliefs about various issues.

Click >> to start

**Modern art paintings manipulation**

***Structured paintings condition (Vasarely):***

**The first part: Evaluating Modern Art Paintings**

You will see a total of nine paintings by a modern art painter. All paintings are by the same artist. This artist is well known for his regular design and alignment of figures.

For each painting, your task is to briefly look at it, form an opinion of it, and answer three questions about the painting. You do not have to like or dislike a particular painting. There are no right or wrong answers. Just feel free to give us your honest opinion about each painting.

Try not to think too long about each painting; what matters most is your first impression.

On the next screen, you will start with the first painting.

*After this, nine paintings followed, which are displayed below. After each painting we asked the following questions:*

- How ugly or beautiful do you find this painting? (1 = *very ugly*, 7 = *very beautiful*)
- How familiar are you with this painting? (1 = *never seen before*, 7 = *very familiar*)
- To what extent do you see a pattern in this painting? (If you only see random strokes of paint, answer “1”; if you clearly see a pattern, answer “7”) (1 = *not at all*, 7 = *very much*).

*Here are the Vasarely paintings that we used:*


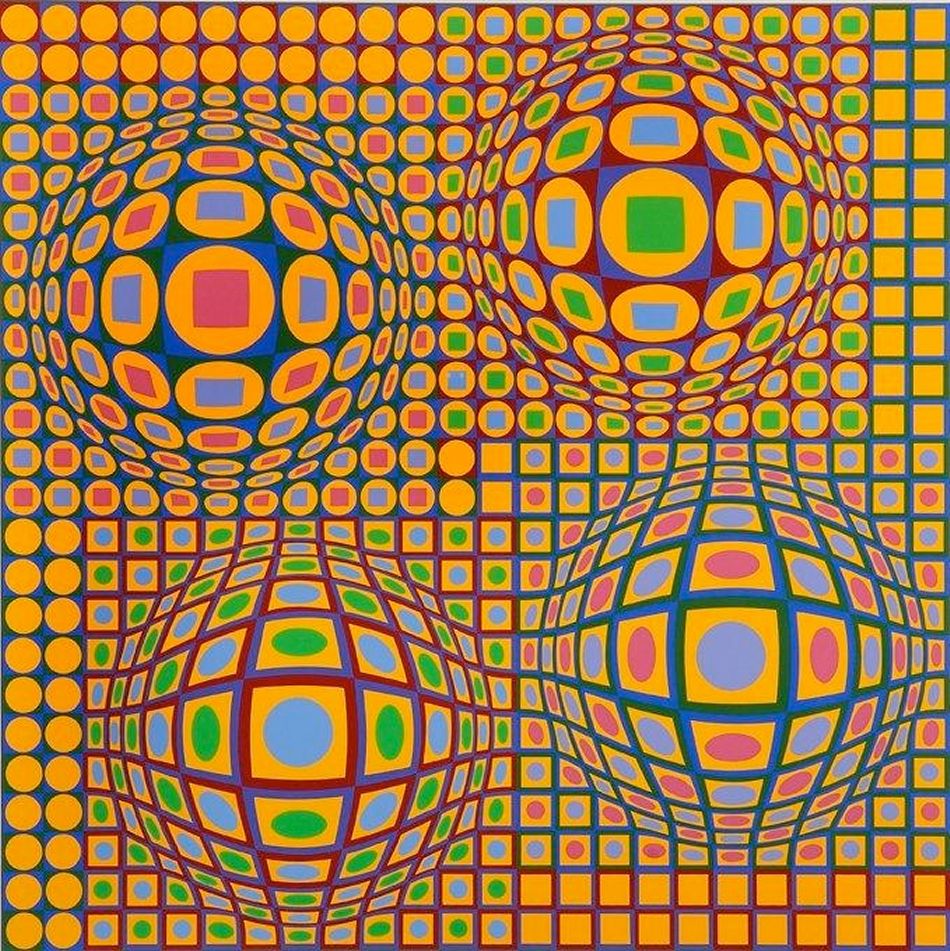


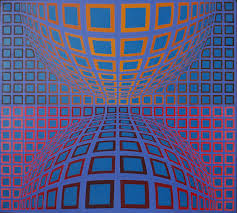


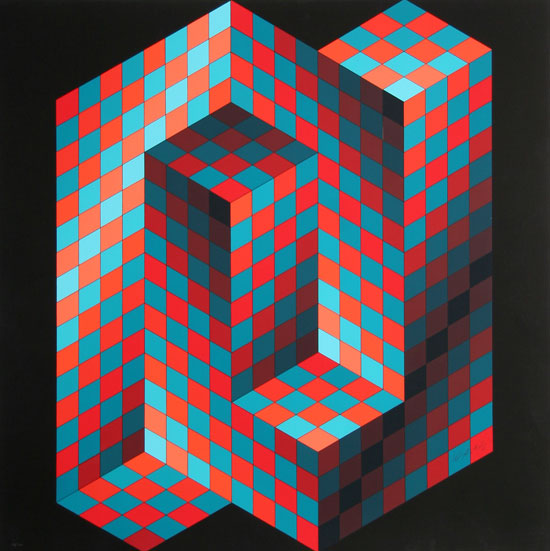


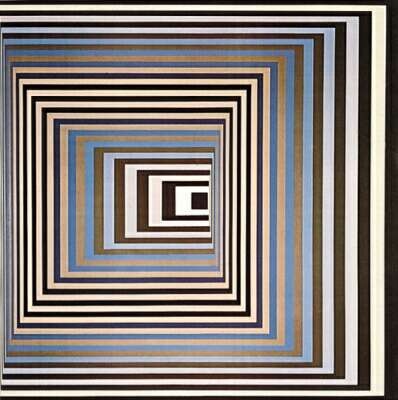


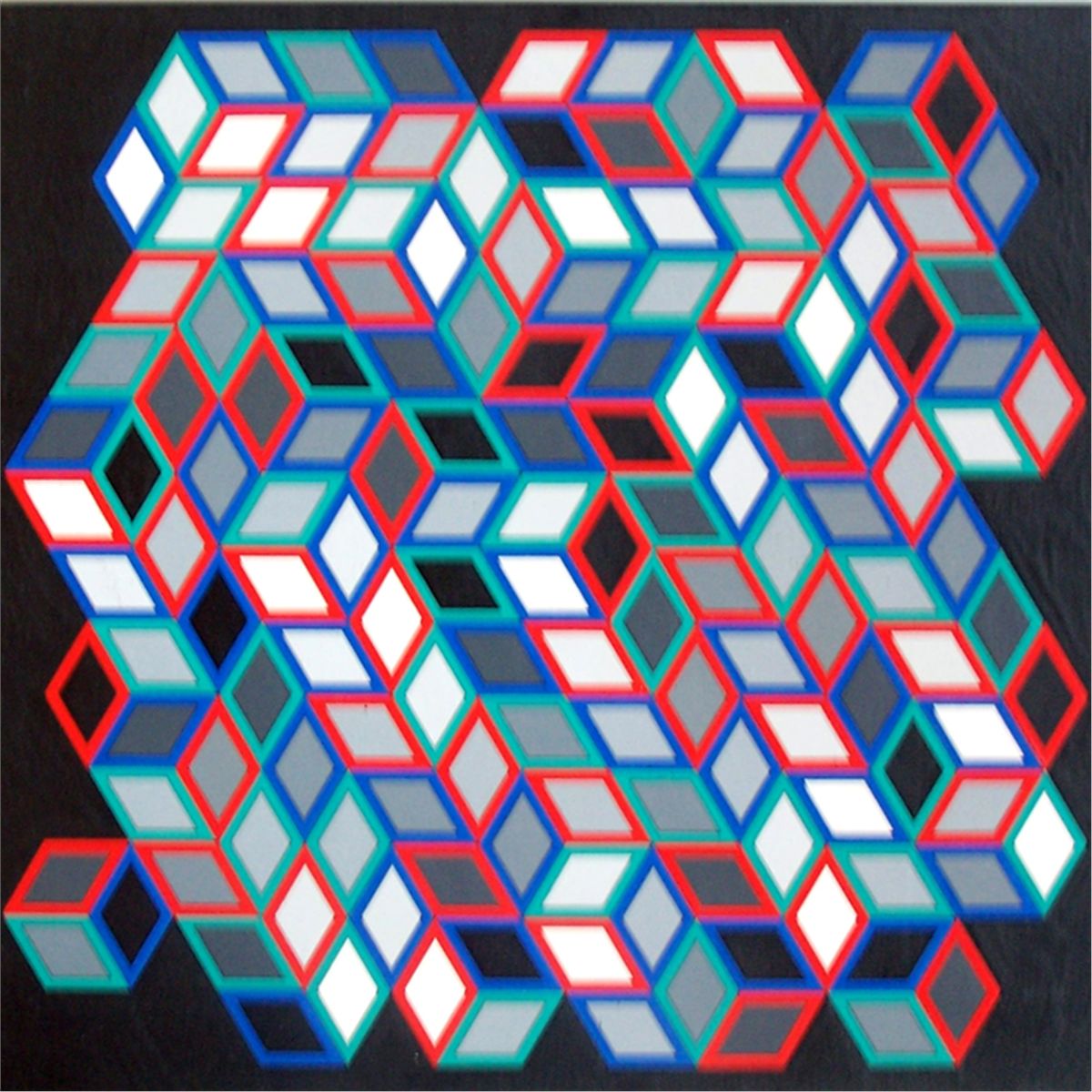


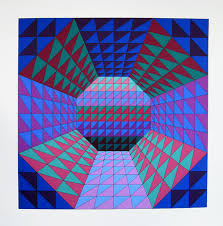


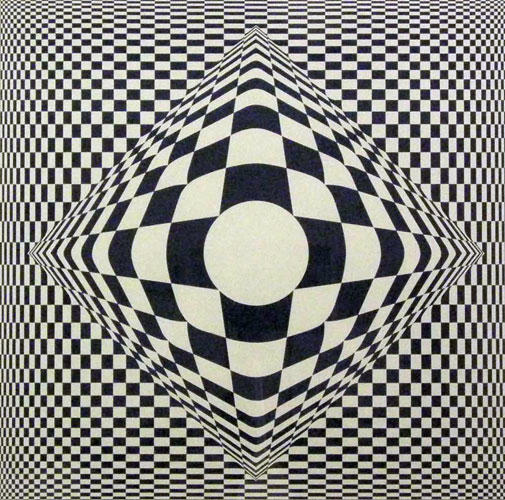


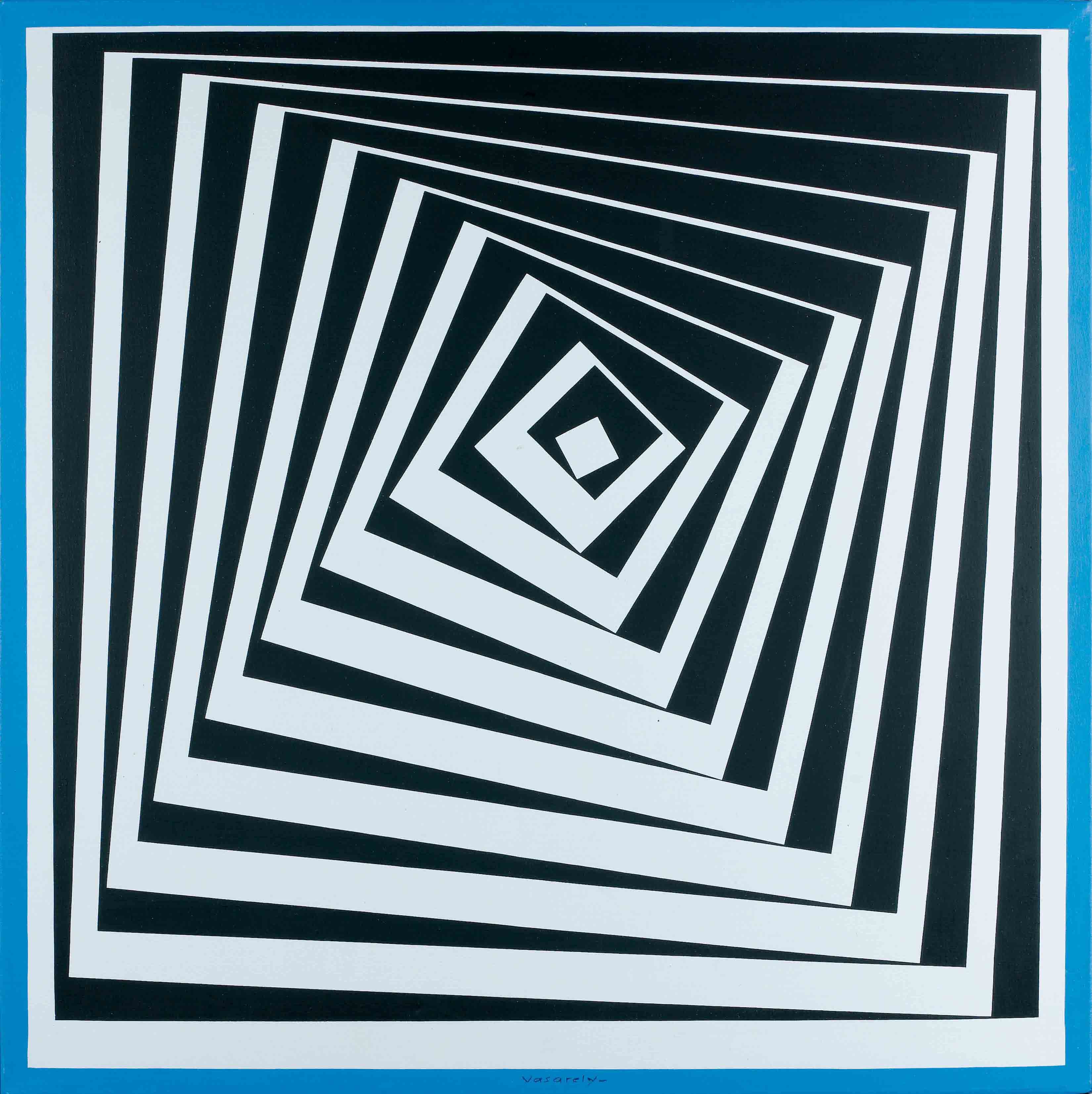


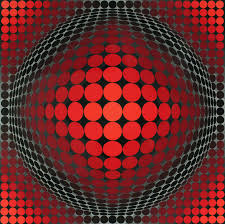


***Unstructured paintings condition (Pollock):***

**The first part: Evaluating Modern Art Paintings**

You will see a total of nine paintings by a modern art painter. All paintings are by the same artist. This artist is well known for his random brush strokes and irregular figures.

For each painting, your task is to briefly look at it, form an opinion of it, and answer three questions about the painting. You do not have to like or dislike a particular painting. There are no right or wrong answers. Just feel free to give us your honest opinion about each painting.

Try not to think too long about each painting; what matters most is your first impression.

On the next screen, you will start with the first painting.

*After this, nine paintings followed, which are displayed below. After each painting we asked the following questions:*

- How ugly or beautiful do you find this painting? (1 = *very ugly*, 7 = *very beautiful*)
- How familiar are you with this painting? (1 = *never seen before*, 7 = *very familiar*)
- To what extent do you see a pattern in this painting? (If you only see random strokes of paint, answer “1”; if you clearly see a pattern, answer “7”) (1 = *not at all*, 7 = *very much*).

*Here are the Pollock paintings that we used:*


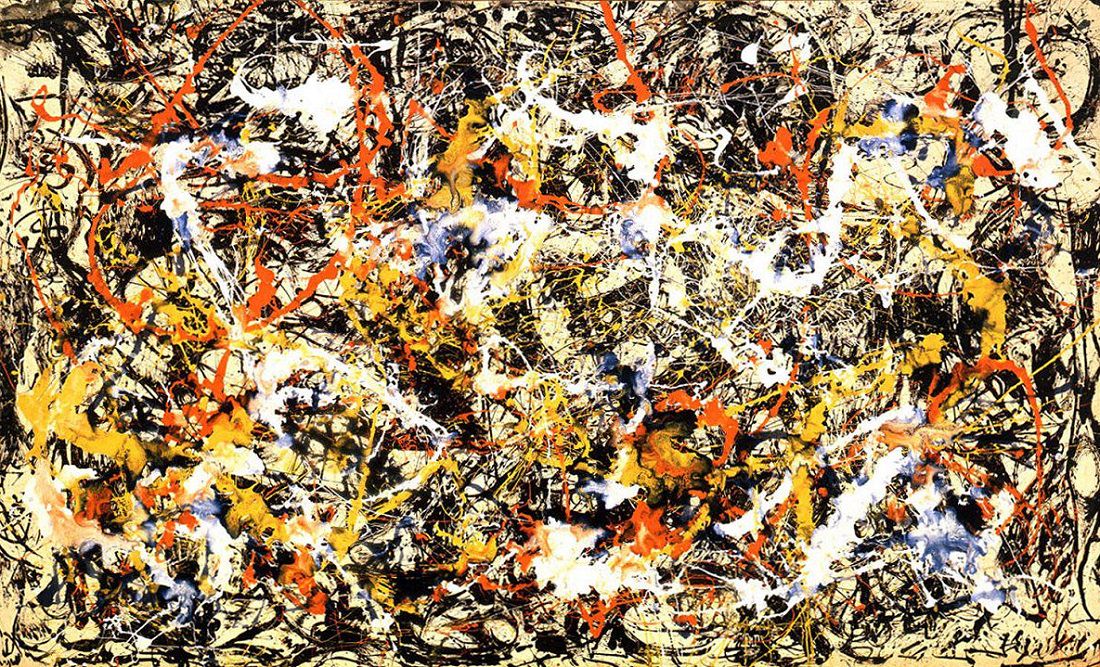


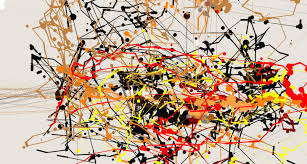


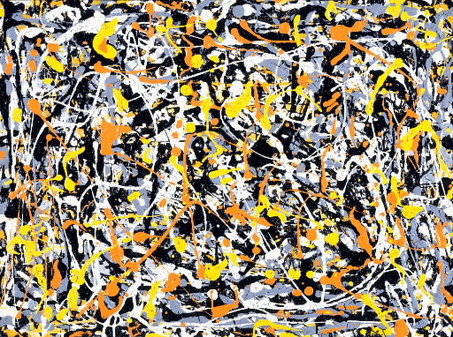


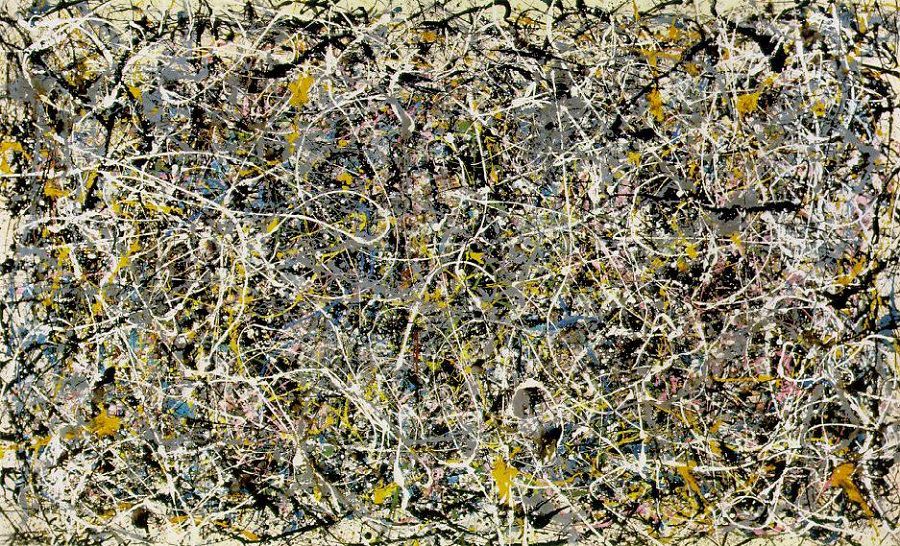


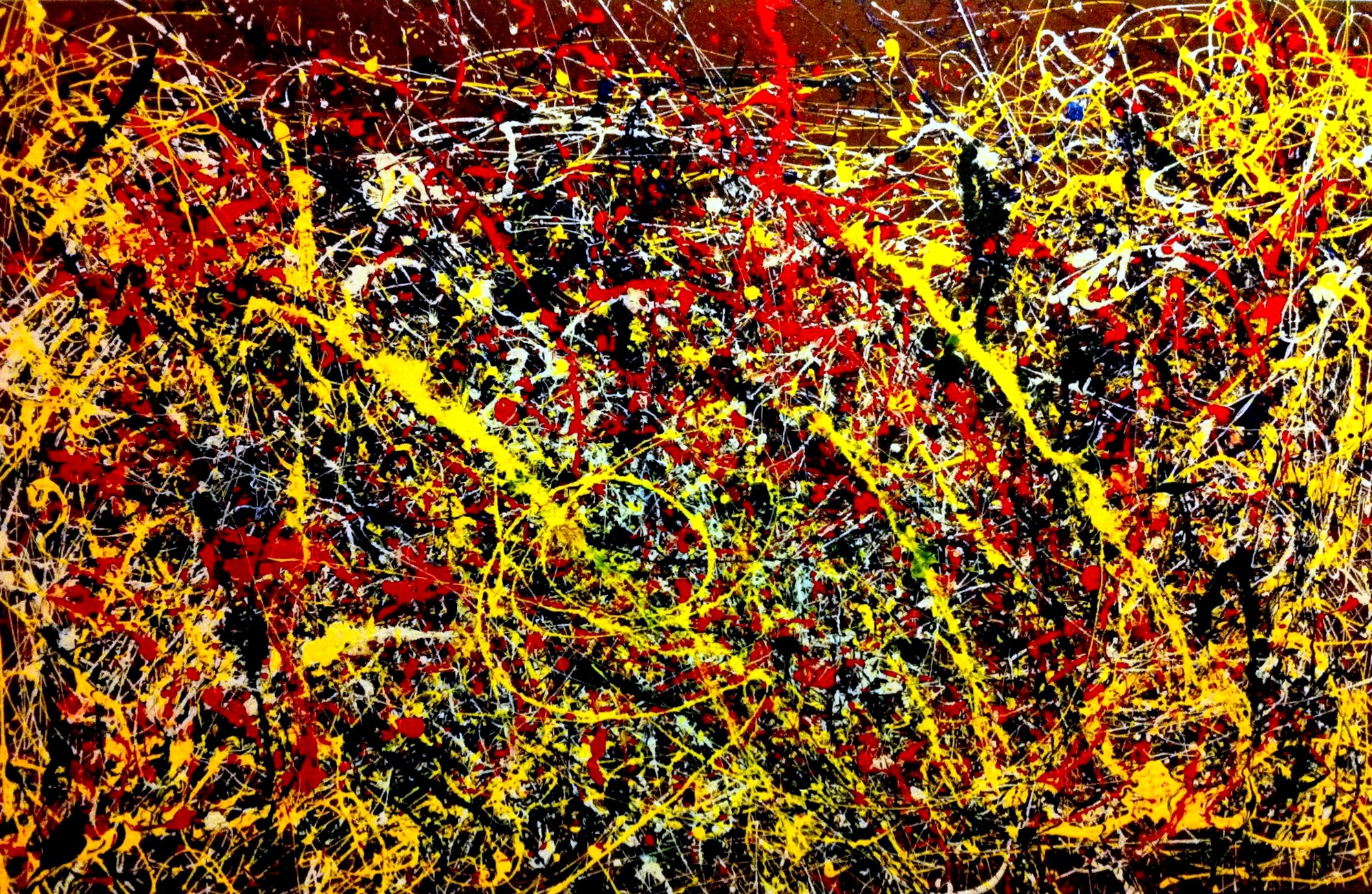


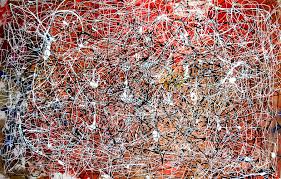


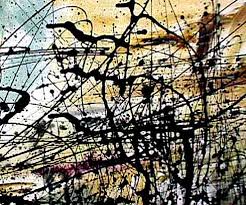


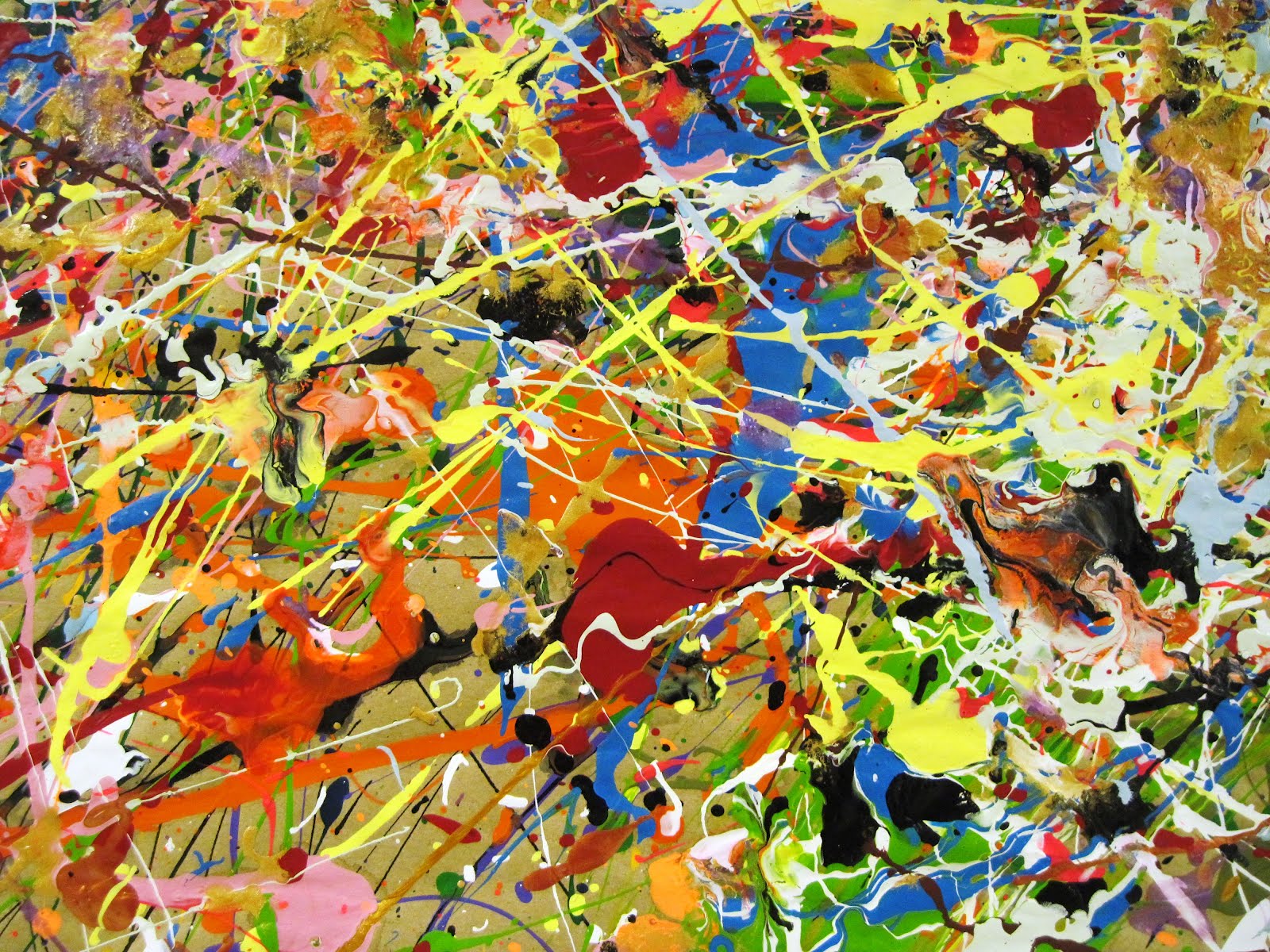


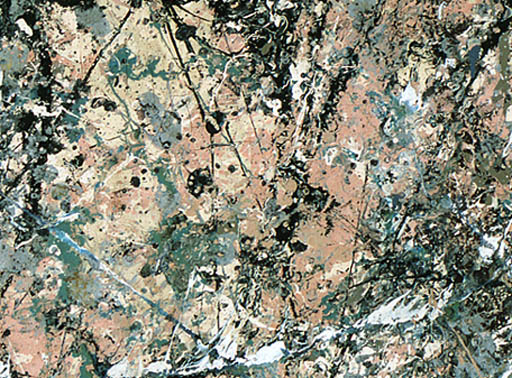


**Measures:**

- *Mood was measured as in Study 2*
- *Existing conspiracy beliefs, fictitious conspiracy beliefs, and supernatural beliefs were measured as in Studies 1 and 2.*

**Study 4**

**Welcome to this Study!**

This study consists of two parts. You will now start with the first part.

On the next page, you will read an excerpt from someone's own Internet blog. Please read the excerpt carefully, you will be asked a few questions about it.

***Supernatural condition:***

**Now, please carefully read the following excerpt from an Internet blog:**

“I believe that there are hidden forces of nature that people do not understand yet, and that determine many important events in life. So often, I think of a particular person, and then that person suddenly calls me. Sometimes too, I meet a stranger that I have never seen before, but yet I somehow feel that I know the person. There is an unknown, inexplicable energy all around us that makes us experience events, realize important insights, or meet certain people at a particular time. I strongly believe that a lot of what we see, hear, think, or do is not a coincidence, but the result of mysterious forces that are all around us”

- Do you agree with the writer? (1 = *Not at all,* 5 = *Very much*)

**Now, please briefly describe a situation that you saw, heard of, or experienced that made you believe that hidden forces of nature were at work.**

***Conspiracy condition:***

**Now, please carefully read the following excerpt from an Internet blog:**

“I believe that there are hidden organizations that influence citizens’ lives in ways that people do not understand, and that explain many events that occur in society. So often, bad things happen to citizens that only benefit a few people at the very top. Sometimes, you can tell that powerful politicians or managers are lying. There are all sorts of unknown and secret organizations that keep track of what citizens are doing, put impactful events in motion, and make sure that we receive certain news at a particular time. I strongly believe that a lot of what we see, hear, think, or do is not a coincidence, but a result of secret organizations that influence us”

- Do you agree with the writer? (1 = *Not at all,* 5 = *Very much*)

**Now, please briefly describe a situation that you saw, heard of, or experienced that made you believe that it was the work of a secret organization.**

***Skeptic condition:***

**Now, please carefully read the following excerpt from an Internet blog:**

“I do not believe in mysterious natural forces or secret organizations. Although we may not always know everything, by and large people have a good sense of how the world works. When I think of a particular person, and then that person suddenly calls me, this is simply a co-incidence. Considering the number of people we think of in one day, it is not even surprising. Sometimes too, bad things happen to citizens, but in the end we need to realize that accidents do happen, and often these are nobody’s fault. I strongly believe that people just make too much out of these sort of things, and that a lot of what we see, hear, think, or do, is the result of nothing more than a coincidence.”

- Do you agree with the writer? (1 = *Not at all,* 5 = *Very much*)

**Now, please briefly describe a situation that you saw, heard of, or experienced that you thought was nothing more than a coincidence.**

***Measures:***

- Mood as in the previous studies

***Pattern perception for world events:***

(1 = *strongly disagree*, 7 = *strongly agree*)

- Societal events that seem unrelated frequently are in fact related
- Many things that happen in the world are no coincidence
- There is a grain of truth in the saying that the wings of a butterfly can cause a hurricane elsewhere.

***Other two pattern perception measures:***

- Patterns in paintings – Perceiving patterns in the Jackson Pollock paintings, identical to Study 3.
- Patterns in coin tosses – identical to Study 1.

**Study 5**

**Conspiracy theory manipulation**

***Pro-conspiracy condition:***

**Please read the short excerpt from a recent Internet article discussing the causes of significant international events. 

Please read it carefully because you will be asked to answer some questions about it.**

**ARTICLE EXCERPT**

Should we be suspicious of government operations?

        For example, did the United States government orchestrate the 9/11 attacks on the Twin Towers? Do the federal authorities routinely collect data on phone calls, emails and other electronic traffic that Americans generate, regardless of whether they have any bearing on a counterterrorism investigation? Questions such as these are widespread in the media and on the Internet, but should we pay any attention to them?

The answer is YES. There are many good reasons to question official accounts.

        To take the example of the National Security Agency’s surveillance programs, the American Civil Liberties Union (ACLU) filed a lawsuit against the NSA, challenging the constitutionality of the NSA telephone call metadata collection program. The Fourth Amendment does not prohibit surveillance, it prohibits surveillance without judicial oversight and clear limits. An order to collect *all* phone records clearly violates this. The Guardian reported that the NSA reads the content of 2,000 e-mail messages and Internet chats every month, and that most of these messages are sent by people who are not communicating about terrorist plots. The NSA is collecting information about every American’s conversations, including who they speak to, or text, for how long, and from where. The government desperately wanted to keep this practice a secret. Perhaps unsurprisingly therefore, a recent survey by the Pew Research Center and the Washington Post of 1,004 adults showed that an overwhelming majority of Americans say the NSA’s program tracking the telephone records of millions of Americans is an unacceptable way for the government to investigate terrorism. Likewise, the significant majority says that the government should not be able to monitor everyone’s email and other online activities.

       The evidence to support alternative accounts for governmental activities is not restricted to the example of government surveillance. To give another example, the US government has been linked to the 9/11 attacks. It is said that there was advance knowledge of the attacks among high-level government officials.  Indeed, there are many inconsistencies in the official accounts and the current ongoing inquests are still uncovering new information that casts these accounts into serious doubt. For example, judicial investigations have shown that in the months leading up to the 9/11 attacks, the CIA repeatedly ignored warnings about a large scale imminent attack on American citizens.

Over the years, many governments have been implicated in major social events. For example, it is argued that the British government was involved in the death of Princess Diana. Indeed, there is evidence to support this claim…*[article continues]*

***Anti-conspiracy condition:***

**Please read the short excerpt from a recent Internet article discussing the causes of significant international events. 

Please read it carefully because you will be asked to answer some questions about it.**

**ARTICLE EXCERPT**
 

Should we be suspicious of government operations? 

         For example, did the United States government orchestrate the 9/11 attacks on the Twin Towers? Do the federal authorities routinely collect data on phone calls, emails and other electronic traffic that Americans generate, regardless of whether they have any bearing on a counterterrorism investigation? Questions such as these are widespread in the media and on the Internet, but should we pay any attention to them?

The answer is NO. There are very few reasons to question official accounts.

         To take the example of the National Security Agency’s surveillance programs, the American Civil Liberties Union (ACLU) filed a lawsuit against the NSA, challenging the constitutionality of the NSA telephone call metadata collection program. However, there is no evidence at all to suggest that the American government monitors ordinary citizens indiscriminately, without adequate oversight and targeting. Specifically, the NSA mines the huge amount of data it gathers daily for patterns that might indicate suspicious activity — calls to a country or a specific person overseas, or unusual communication behavior, like a spike in calls, that coincides with what the NSA is seeing elsewhere. A computer algorithm flags potentially suspicious numbers, giving investigators justification to demand wiretaps from a federal judge. Electronic data sweeps are performed to ensure citizens’ safety. In fact, it is through this electronic traffic that the NSA has been able to recognize and prevent attacks before they even happen, over fifty times since 9/11. Perhaps unsurprisingly therefore, a recent survey by the Pew Research Center and the Washington Post of 1,004 adults showed that an overwhelming majority of Americans support the NSA’s surveillance efforts. Moreover, Americans’ views of the government’s monitoring of electronic traffic to prevent possible terrorism are largely unchanged since 2002, shortly after the 9/11 terrorist attacks.

         The lack of evidence to support alternative accounts for governmental activities is not restricted to the example of government surveillance. To give another example, the US government has been linked to the 9/11 attacks. It is said that there was advance knowledge of the attacks among high-level government officials. However, there is no evidence to support this account and the current ongoing inquests are revealing only information that is consistent with the official statements. For example, the inquests/investigations recently ruled out any American intelligence involvement in the attacks.

        Over the years, many governments have been implicated in major social events. For example, it is argued that the British government was involved in the death of Princess Diana. However, there is no evidence to support this claim…*[article continues]*

**Measures:**

***Manipulation check:***

(1 = *not at all*, 7 = *very much*):

- Is there reason for concern about the NSA security programs?
- Is there reason to think that the NSA listens to private phone conversations that are unrelated to terrorist plots?
- Is there reason to think that the NSA reads the content of e-mail and Internet chat messages that are unrelated to terrorist plots?
- Is there reason to be suspicious about other governmental operations besides the NSA’s surveillance programs?

***Mood:***

*This was measured as in the previous studies*

***Pattern perception for world events:***

(1 = *strongly disagree*, 7 = *strongly agree*)

- Societal events that seem unrelated frequently are in fact related
- Many things that happen in the world are no coincidence
- There is a grain of truth in the saying that the wings of a butterfly can cause a hurricane elsewhere.

***Dependent variables:***

*These were the same as in Studies 1 to 3.*
